# Long intergenic non-coding RNA *APOC1P1-3* inhibits apoptosis by decreasing *α*-tubulin acetylation in breast cancer

**DOI:** 10.1038/cddis.2016.142

**Published:** 2016-05-26

**Authors:** X-H Liao, J-G Wang, L-Y Li, D-M Zhou, K-H Ren, Y-T Jin, L Lv, J-G Yu, J-Y Yang, Q Lu, Q Zou, J Yu, X-P Liu, P Zhou

**Affiliations:** 1 Department of Physiology and Pathophysiology, School of Basic Medical Sciences, Fudan University, No. 138 Yixueyuan Road, Shanghai, China.; 2 Department of Pathology, School of Basic Medical Sciences, Fudan University, No. 138 Yixueyuan Road, Shanghai, China; 3 Department of Breast Surgery, Huashan Hospital, Fudan University, No. 12 Urumqi Middle Road, Shanghai, China; 4 Department of Medicine, University of Louisville, Louisville, KY 40292, USA; 5 Department of Pathology, The Fifth People's Hospital of Shanghai, Fudan University, No. 128 Ruili Road, Shanghai, China

## Abstract

Increasing evidence indicates that long non-coding RNAs (lncRNAs) act as important regulatory factors in tumor progression. However, their roles in breast cancer remain largely unknown. In present studies, we identified aberrantly expressed long intergenic non-coding RNA *APOC1P1-3* (*lincRNA-APOC1P1-3)* in breast cancer by microarray, verified it by quantitative real-time PCR, and assessed methylation status in the promoter region by pyrosequencing. We also investigated the biological functions with plasmid transfection and siRNA silencing experiments, and further explored their mechanisms by RNA pull-down and RNA immunoprecipitation to identify binding proteins. We found that 224 lncRNAs were upregulated in breast cancer, whereas 324 were downregulated. The *lincRNA-APOC1P1-3* was overexpressed in breast cancer, which was related to tumor size and hypomethylation in its promoter region. We also found that *APOC1P1-3* could directly bind to tubulin to decrease *α*-tubulin acetylation, to inactivate caspase-3, and to inhibit apoptosis. This study demonstrates that overexpression of *APOC1P1-3* can inhibit breast cancer apoptosis.

Long non-coding RNAs (lncRNAs) are a group of non-protein-coding transcripts longer than 200 nucleotides. They are found in sense or antisense orientation to protein-coding genes, within introns of protein-coding genes or in intergenic regions of the genome. Although significant numbers of lncRNAs have been identified, most of them remain largely uncharacterized and little is known about their functions.^1^ There are reports that they not only interact directly with DNA, mRNAs or proteins (such as transcription factors), but also with other regulatory non-coding RNAs.^2^ By binding to regulatory components and forming lncRNA–gene complexes, they cause genetic regulations or epigenetic modifications.^3^

Recently, lncRNAs draw attention on their potential contribution towards disease etiology. Accumulating findings implicate that lncRNAs are expressed aberrantly in the cancer development process, including proliferation, metastasis, and apoptosis. For example, *lncRNA-GAS5* expression is significantly downregulated in breast cancer cells, promoting apoptosis.^4^ The long intergenic non-coding RNA (lincRNA) *p21*, which contains p53-binding sites for activation during DNA damage, is regarded as an important repressor in the p53-mediated pathway and apoptosis.^5^ The lncRNAs involved in breast carcinogenesis are still in need of further exploring.

Apolipoprotein C-I pseudogene 1 (*APOC1P1*) is a pseudogene located in 19q13.2 between apolipoprotein C-I and apolipoprotein C-IV. It encodes three RNA transcript variants that belong to lincRNA family. The variant 3 (*lincRNA-APOC1P1-3*), which is shorter than the variant 1 and 2, lacks an alternate internal segment and uses an alternate internal splice site. Its expression and function in human diseases are unknown. In present studies, we tested the hypothesis that *APOC1P1-3* overexpression involved in breast cancer progression. Using the microarray, we confirmed that *lincRNA-APOC1P1-3* is highly expressed in breast cancer tissues. Microarray results were validated with quantitative real-time PCR in breast cancer cell lines and tissues. Biological functions of *lincRNA-APOC1P1-3* were assessed by gain *versus* loss function studies and regulatory mechanisms were investigated by RNA pull-down, RNA immunoprecipitation (RIP), and pyrosequencing. Our data support this hypothesis.

## Results

### LincRNA-APOC1P1-3 is overexpressed in breast cancers

**Figure 1:**
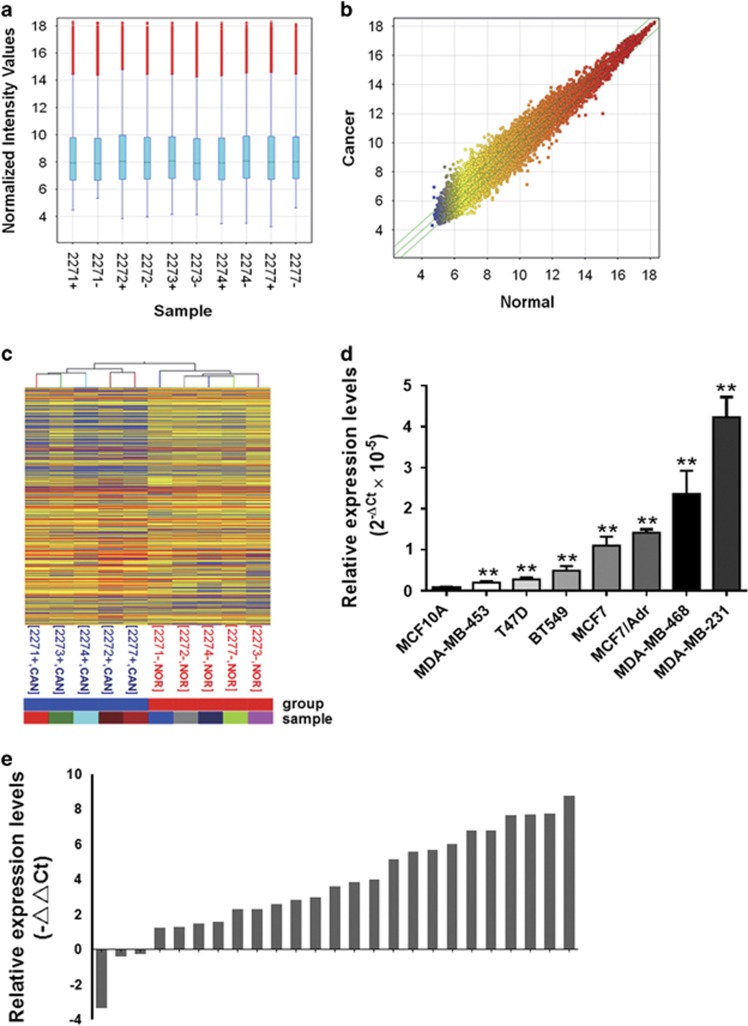
LncRNA microarray screening and qPCR validation for differentially expressed lncRNAs in breast cancer. The microarray results in five pairs of fresh breast cancer tissues and corresponding para-cancer normal tissues were shown in (**a**, **b**, and **c**). (**a**) Quality assessment of lncRNA data after filtering using box plot. The box plot is a convenient way to quickly visualize the distributions of a data set. It is commonly used for comparing the distributions of the intensities from all samples. After normalization, the distributions of log2 ratios among all tested samples are nearly the same. Red bars indicate abnormal values. Blue boxes, the bottom and top of the box are the first and third quartiles, and the band inside the box is the median. (**b**) The scatter plot for assessing the lncRNA expression variation between cancer and para-cancer tissues. The values of *X* and *Y* axes in the scatter plot are the normalized signal values of the samples (log2 scaled) or the averaged normalized signal values of groups of samples (log2 scaled). The green lines are fold change lines (the default fold change value given is 1.5). The lncRNAs above the top green line and below the bottom green line indicated >1.5-fold change of lncRNAs between the two compared samples or the two compared groups of samples. (**c**) Hierarchical clustering for ‘differentially expressed lncRNAs for cancer *versus* para-cancer'. ‘Red' indicates high relative expression, and ‘blue' indicates low relative expression. The result from hierarchical clustering shows a distinguishable lncRNA expression profiling among samples. (**d**) qPCR detection showed all breast cancer cell lines bear higher expression level of *APOC1P1-3* than non-tumoral mammary epithelial cell line MCF10A. Data are shown as the mean±S.D. Error bars indicate S.D. **P<0.01 *versus* control (MCF10A). (**e**) qPCR detection in 25 pairs of fresh tissues showed *APOC1P1-3* was highly expressed in breast cancer tissues. ΔΔCt=ΔCt (cancer)−ΔCt (normal), ΔCt=Ct (*APOC1P1-3*)−Ct (*GAPDH*)

Our microarray results (NCBI GEO accession: GSE80266) showed that 224 lncRNAs increased and 324 decreased in breast cancer tissues (fold change ⩾1.5, Supplementary Table S4). Hierarchical clustering showed systematic variations in expression of lncRNAs in normal *versus* cancer tissues (Figures 1a–c). We found that *lincRNA-APOC1P1-3* (fold change=2.02, *P*-value=0.02, and full length=631 bp) met the selection criteria and then was taken into further validation. To investigate the role of *APOC1P1-3*, we compared its expression profiles in cultured cells (MCF10A *versus* BT549, MCF7, MDA-MB-231, MDA-MB-453, MDA-MB-468, MCF7/Adr, and T47D) and 25 pairs fresh tissues (cancer *versus* matched normal tissues) with qPCR. Again, our data showed *lincRNA-APOC1P1-3* was overexpressed in both breast cancer cell lines and tissues (Figures 1d and e).

### Hypomethylation in APOC1P1 promoter region

**Figure 2:**
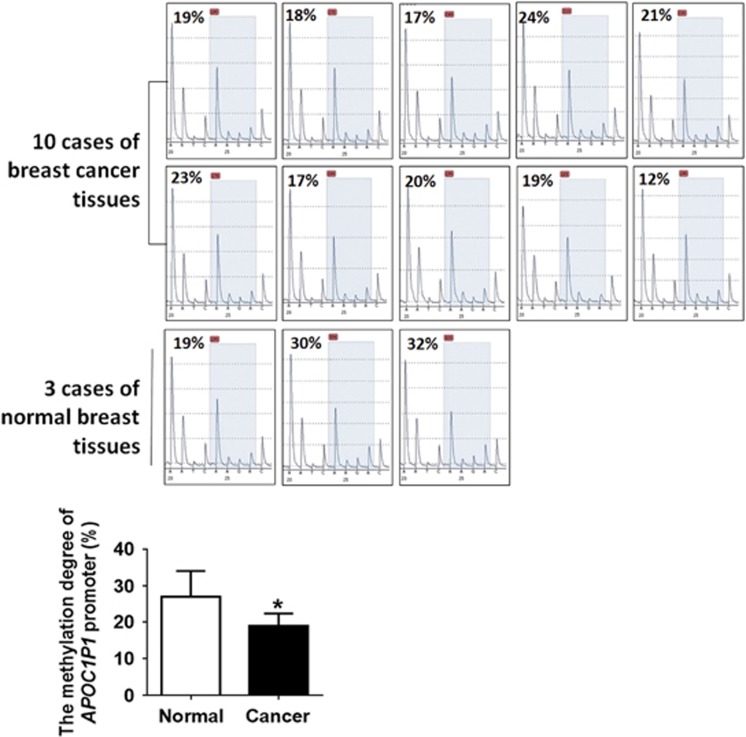
The methylation degree of *APOC1P1-3* promoter region. The methylation degree of breast cancer is lower than the normal mammary tissues (*P<0.05). Images are the representation of statistically different site. Data are shown as the mean±S.D. Error bars indicate S.D.

Methylation of gene promoter has been proved to be eventful in gene epigenetic regulation. To determine whether methylation modifications exist in *APOC1P1* gene promoter region, we quantified C/G methylation levels in the first exon of *APOC1P1* with its upstream 1000-bp region using pyrosequencing in 3 normal and 10 breast tissues to quantify the degree of methylation at each CG site. All 16 C/G sites of the designated region were subjected to pyrosequencing. The pyrosequencing results showed that one of the 16 C/G methylation sites was significantly hypomethylated in breast cancer tissues when compared with normal tissues (Figure 2), whereas the other fifteen sites showed no differences. These results indicate that the hypomethylation of the C/G site may contribute to the upregulation of *APOC1P1* in breast cancer.

### LincRNA-APOC1P1-3 is related to the tumor size

To characterize the role of *APOC1P1-3* overexpression in breast cancers, we examined the relationship between expression of *APOC1P1-3* and clinicopathologic parameters (age, molecular subtypes (luminal A like, luminal B like, HER2 positive, and triple negative),^7^ breast cancer biomarkers (estrogen receptor (ER), progesterone receptor (PgR), and HER2), lymph node status, distant metastasis, and pTNM stage). We found that *APOC1P1-3* expression was positively associated with tumor size (*P*=0.0142). Tumors with a larger volume (⩾2.5 cm) tended to exhibit higher *APOC1P1-3* expression. However, there was no significant relationship between *APOC1P1-3* expression and other parameters (Table 1).

### LincRNA-APOC1P1-3 regulates early apoptosis in breast cancer cells

**Figure 3:**
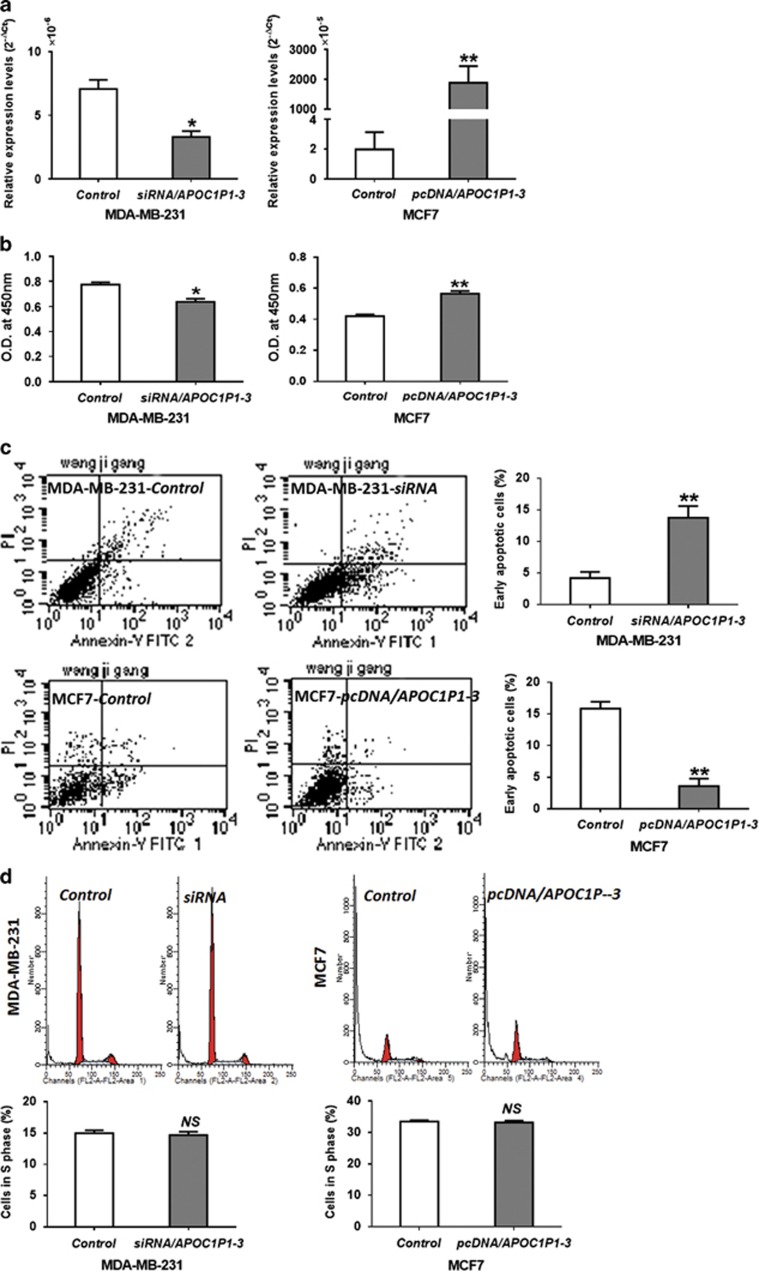
*APOC1P1-3* can affect apoptosis of breast cancer cells. (**a**) qPCR to detect transfection efficiency. (**b**–**d**) After transfection for 24 h, CCK8 assay and flow cytometry were carried out to detect cell proliferation (**b**), early apoptosis (**c**), and cell cycle (**d**). MCF7 cells were cultured in serum-free medium. Data are shown as the mean±S.D. based on at least three independent experiments. Error bars indicate S.D. *P<0.05 *versus* control; **P<0.01 *versus* control; NS, no significant *versus* control

**Figure 4:**
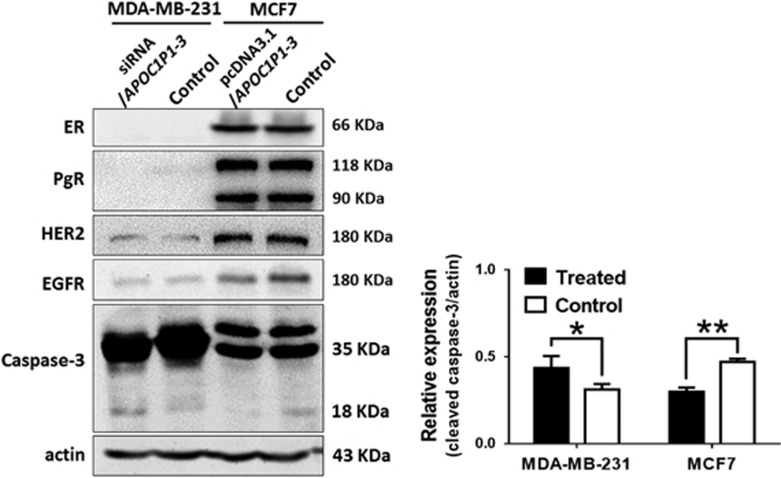
*APOC1P1-3* can affect caspase-3 activation, but can not affect expressions of ER, PgR, HER2, and EGFR. MDA-MB-231 cells were transfected with *siRNA/APOC1P1-3*, and MCF7 cells were transfected with *pcDNA3.1/APOC1P1-3*. MDA-MB-231 cells were negative for ER and PgR, and weakly positive for HER2 and EGFR. MCF7 cells were positive for ER, PgR, HER2, and EGFR. The downregulation of *APOC1P1-3* by siRNA can induce activation of caspase-3, and the upregulation of *APOC1P1-3* by pcDNA can inhibit that. However, the alteration of *APOC1P1-3* expression cannot affect ER, PgR, HER2, and EGFR. SiRNA, *APOC1P1-3* knockdown by siRNA transfection; pcDNA, *APOC1P1-3* overexpression by transfection with pcDNA3.1 plasmid encoding *APOC1P1-3*. *P<0.05 *versus* control; **P<0.01 *versus* control

To determine the biological function of *lincRNA-APOC1P1-3*, we performed gain/loss function studies. We found that MCF7 and MDA-MB-231 cells can be effectively upregulated and downregulated by *pcDNA3.1* and *siRNA* transfection, respectively (Figure 3a). The CCK8 proliferation assay showed that viable cells in *siRNA/Control* group and *pcDNA3.1/APOC1P1-3* (*APOC1P1-3* overexpression) group were more than those in *siRNA/APOC1P1-3* (*APOC1P1-3* knockdown) group and *pcDNA3.1/Control* group, respectively (Figure 3b). Further, flow cytometry (for early apoptosis) demonstrated that the upregulation of *APOC1P1-3* inhibited, whereas downregulation induced cell apoptosis (Figure 3c). However, cell cycles were not affected (Figure 3d). Furthermore, we found that alterations of *APOC1P1-3* affected caspase-3 activation, whereas did not affect ER, PgR, HER2, and epithelial growth factor receptor (EGFR) expressions (Figure 4).

### LincRNA-APOC1P1-3 can bind *α*-tubulin and modify its acetylation

**Figure 5:**
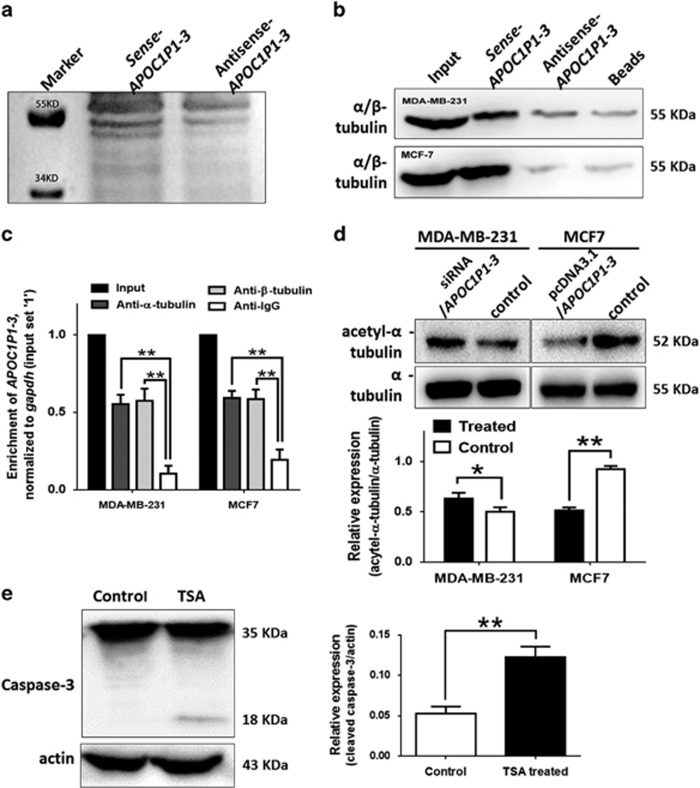
*APOC1P1-3* can bind to tubulin and modify its acetylation levels. (**a**) RNA pull-down to detect the specific combining proteins of *APOC1P1-3*, silver stain of the SDS-PAGE gel showed that there was a specific bond between 34 and 55 kDa. The bond was cut to mass spectrometry analysis and was identified as tubulin. (**b**) Western blot to validate the mass spectrometry results in MDA-MB-231 and MCF7 cell lines. (**c**) Relative RIP experiments were performed with anti-tubulin antibodies on extracts from MDA-MB-231 and MCF7 cells, respectively, with IgG as a negative control. The purified RNA was used for qPCR analysis, and the enrichment of *APOC1P1-3* was normalized to input. *P<0.05 *versus* control. (**d**) The *α*/*β*-tubulin and acetylated *α*-tubulin were detected using western blot. The total proteins of both *α*-tubulin and *β*-tubulin were not changed, and the acetylated *α*-tubulin was decreased in *APOC1P1-3* overexpressed groups. (**e**) Trichostatin A TSA induced caspase-3 activation in MDA-MB-231 cells. Data are shown as the mean±S.D. based on at least three independent experiments. Error bars indicate S.D. *P<0.05 *versus* control; **P<0.01 *versus* control

To investigate the mechanism of *APOC1P1-3*-induced early apoptosis, we determined whether *APOC1P1-3* could bind and interact with apoptosis-related proteins. Therefore, we first conducted RNA pull-down assay in MCF7 cells to determine binding proteins (Figure 5a). The mass spectrometry analysis for the specific band revealed that tubulin was a potential binding protein (Supplementary Table S5). To validate the mass spectrometry result, we performed a western blot using the captured protein from RNA pull-down assays in MDA-MB-231 and MCF7 cells (including *α*-tubulin and *β*-tubulin; Figure 5b). Furthermore, we performed a RIP assay with antibodies for *α*-tubulin and *β*-tubulin, and detected a significant enrichment of *APOC1P1-3* by further qPCR study (Figure 5c).

Suppressed tubulin polymerization and increased *α*-tubulin acetylation contribute to apoptosis of cancer cells.^8, 9^ To further confirm effects of *APOC1P1-3* on acetylation levels of *α*-tubulin and attenuation of apoptosis, we examined acetylated *α*-tubulin levels in MCF7 and found that exogenous expression of *APOC1P1-3* significantly reduced acetylated *α*-tubulin (Figure 5d), although the total protein content of *α*/*β*-tubulin were unchanged. Furthermore, acetyltransferase inhibition with Trichostatin A demonstrated that the increasing acetylation of *α*-tubulin induced cell apoptosis (Figure 5e).

## Discussion

**Figure 6:**
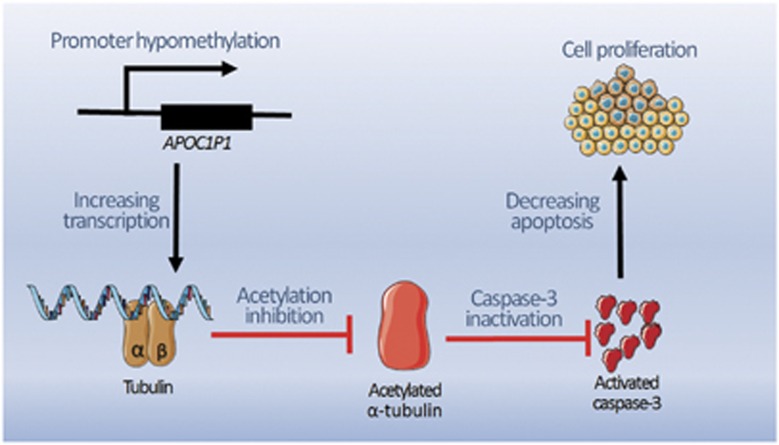
The regulating mechanism of *APOC1P1-3* in breast cancer. Our study demonstrates that *lincRNA-APOC1P1-3* is overexpressed in breast cancer. The promoter region of *APOC1P1* is hypomethylated, which contributes to transcription activation and *APOC1P1-3* overexpression. *LincRNA-APOC1P1-3* can bind to tubulin, and then increase *α*-tubulin acetylation and inhibit apoptosis

Identification of lncRNA is one of the most significant discoveries in contemporary science. LncRNAs have an essential role in epigenetics,^10^ transcriptional regulation, growth and development,^11^ and constitute part of the nucleus.^12^ LncRNAs also function in tumor cell proliferation, apoptosis, invasion, and metastasis.^13^ In current studies, we found *lincRNA*-*APOC1P1-3* was overexpressed in breast cancer and the promoter region was hypomethylated. *APOC1P1-3* could bind to *α*-tubulin and affect its acetylation, leading to cell apoptosis inhibition. On the basis of these findings, we propose a regulatory mechanism for *APOC1P1-3* in breast cancer (Figure 6).

**Figure 7:**
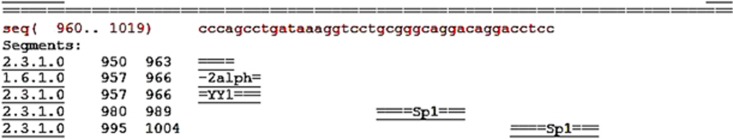
Transcription factor Sp1 is a potential binding protein of the hypomethylation region of *APOC1P1*. AliBaba 2.1 (http://www.gene-regulation.com/pub/programs/alibaba2/index.html) was used to predict the binding proteins. The promoter sequence and the binding segments of Sp1 are shown

Methylation of gene promoter is important in gene epigenetic regulation. Hypomethylation of lncRNA has been found in cancers.^14, 15^ Some breast cancer related genes, such as *BRCA1*,^16^ are known to be regulated by methylation modification. Recently, the methylation of lncRNA was also reported.^17^ As expression of *APOC1P1-3* is high in breast cancer, using pyrosequencing to detect the CpG methylation levels, we examined whether the promoter region was hypomethylated. Cross talk occurs between lncRNAs and methylation regulatory network. Presence of CpG island demethylation in the lncRNA promoter leads to overexpression of lncRNA transcription.^18^ Our study suggests that the hypomethylation of lncRNA promoter regulates expression of lncRNA. We have predicted binding proteins of the hypomethylation region using AliBaba 2.1, which suggests transcription factor Sp1 is a potential binding protein (Figure 7). In view of the important regulatory role of Sp1 in gene expression, we consider overexpression of *APOC1P1-3* may be due to the promoter hypomethylation followed by Sp1 activation. Further investigation on involvements of histone demethylases and Sp1 is needed.

LncRNAs affect tumor proliferation via cell cycle and apoptosis.^13^ Caspase-3 is the main executor of apoptosis. Expression levels of cleaved caspase-3 reflect caspase-3 activities and degrees of apoptosis. Thus, we assessed early apoptosis and caspase-3 activation (CCK8 assay, flow cytometry, and western blot analysis) during *APOC1P1-3* silencing and overexpression. We found that *APOC1P1-3* repressed apoptosis of breast cancer to facilitate its proliferation through altering the apoptotic protein levels. These results support that *APOC1P1-3* regulates the breast cancer development by regulating apoptosis.

In spite of the complexity and diversity of mechanisms, most studies report that lncRNAs exert effects by directly binding to chromatin modification complexes (*HOTAIR*, *Xist*, and *Tsil*)^19, 20, 21^ or non-chromatin modification proteins (Dreh).^22^ We used an RNA pull-down assay to identify binding proteins. Mass spectrometry, western blots, and RIP identified tubulin as a specific binding protein. Tubulin is the major constituent of microtubules and cytoskeletal structure, and has critical role in cell mitosis and chromosome segregation, as well as cell proliferation and migration.^23^ Post-translational modifications of *α*- and *β*-tubulin are key in regulation.^24, 25, 26^ *α*-Tubulin acetylation (transfer of the acetyl group from acetyl-coenzyme A to Lys-40) regulates the structure and function of microtubules.^27^ Inhibition of tubulin polymerization and increased acetylation of *α*-tubulin contribute to cancer cell apoptosis.^8, 9^ We found that *APOC1P1-3* bound to tubulin, and *APOC1P1-3* overexpression decreased *α*-tubulin acetylation, supporting that tubulin may be a target of *APOC1P1-3.* However, the effects of *APOC1P1-3* on acetylation of *α*-tubulin remains unknown.

Apolipoprotein C-1 (APOC1) protein is highly expressed in pancreatic cancer. It stimulates cell proliferation and prevents cell apoptosis.^28^ However, APOC1 protein was found to be downregulated in breast cancer patients.^29^ *APOC1P1* is the pseudogene of *APOC1.* Generally, the antisense transcripts produced from pseudogenes can hybridize to corresponding mRNAs, forming dsRNAs cleaved by Dicer to endogenous siRNAs.^30^ Our findings provide an explanation for low expression levels of APOC1 in breast cancer patients. Further study is clearly needed to investigate the interaction between the two genes.

In summary, our study demonstrates that *lincRNA-APOC1P1-3* is overexpressed in breast cancer, and its upregulation promotes cell proliferation by suppressing cell apoptosis. *APOC1P1-3* can bind to tubulin, and then increase *α*-tubulin acetylation and inhibit apoptosis. In addition, the promoter region of *APOC1P1* is hypomethylated, which contributes to the transcription activation and *APOC1P1-3* overexpression. We conclude that *lincRNA-APOC1P1-3* is involved in the breast cancer development.

## Materials and Methods

The information for tumor tissues, cell lines, PCR, western blot, immunohistochemistry, proliferation assay, and cell cycle assay were provided in the Supplementary Materials.

### LncRNA expression microarray analysis

Five matched breast cancer and normal tissues were used for microarray (Table S1). Total RNA was extracted using TRIzol (Ambion, Carlsbad, CA, USA), and transcribed into fluorescent cDNA using Quick Amp Labeling kit (Agilent, Palo Alto, CA, USA). After hybridization, using Human LncRNA Microarray v2.0 (Arraystar, Rockville, MD, USA), slides were scanned with the Agilent DNA Microarray Scanner (Agilent p/n G2565BA) and analyzed with Agilent Feature Extraction software v. 11.5.1.1. Quantile normalization and subsequent data processing were performed using the Agilent GeneSpring GX v11.5.1. Differentially expressed lncRNAs with statistical significance were identified through volcano plot filtering (threshold: *P*-value ⩽0.05, fold change ⩾1.5, and false discovery rate ⩽0.05). Microarray array data analysis was completed by Shanghai KangChen bio-tech (Shanghai, China). The candidate lncRNAs should meet the following criteria: (1) RNA length <3 kb; (2) negative X-hybrization (cross-hybridization) result: the probe can not be hybridized with other lncRNAs or mRNAs; (3) sequences do not overlap with nearby mRNAs; (4) *P*-value, as small as possible; fold change, as big as possible; and raw intensity, as high as possible; and (5) comparable with the latest version of the relative database (NCBI Reference Sequence, UCSC Knowngenes, and Ensembl Genome).

### Pyrosequencing

The pyrosequencing work was accomplished by the cpgbiotech company (Shanghai, China). Three normal breast tissues and 10 breast cancer tissues were obtained from Huashan Hospital, Fudan University. Primers were designed by PyroMark Assay Desigen Software 2.0 (Qiagen, Hilden, Germany). Amplification primers sequences (in 5′–3′ orientitation) and the sequencing primers are listed in the Supplementary Table S2. One of the primers must be biotinylated, which enables conversion of the PCR product to a single-stranded DNA template for pyrosequencing. The technological processes were: (1) bisulfite treatment and elution of genomic DNA (C→U and mC→mC; Qiagen); (2) PCR amplification (U→T and mC→C; PyroMark PCR kit, Qiagen, Hilden, Germany). Both methylated and unmethylated DNA sequences of the designated regions were amplified with its specific primers; (3) streptavidin-coated beads separated specific PCR products into single strand; (4) sequencing primer was added, which annealed to a fixed single-stranded DNA template; and (5) quantitative methylation detection by pyrosequencing was completed with Biotage PyroMark Q24 system (Qiagen, Hilden, Germany) according to manufacturer's instructions, and data were analyzed with PyroMark software (Qiagen). Calculation of C:T peaks represent the methylation.^6^

### Plasmid and siRNA transfection

The cDNA encoded full-length *lincRNA-APOC1P1-3* was PCR-amplified using primers (5′-CAACCAAGCCCTCCAGCAAG-3′ and 5′-GCCTCAGCCTCCCGAATAG-3′), amplification was performed for 35 cycles at 95 °C for 45 s, at 60 °C for 45 s, and at 72 °C for 1 min, and subcoloned into *Bam HI* and *Xho I* sites of a pcDNA3.1 vector (Invitrogen, Carlsbad, CA, USA), named *pcDNA3.1/APOC1P1-3*. Transfections for *pcDNA3.1/APOC1P1-3* and *siRNA/APOC1P1-3* (Supplementary Table S3) were performed using the lipofectamine 2000 (Invitrogen) with Opti-MEM (Gibco, Grand Island, NY, USA) according to the manufacturer's instructions. Total RNA and protein were collected after 24 and 48 h, respectively.

### Apoptosis detection

Flow cytometry was used to detect the apoptotic cells. After transfection with lipofectamine 2000 and Opti-MEM for 6 h, cells were maintained in fresh medium supplemented with 1% FBS for 24 h. Thereafter, cells were collected and washed with phosphate-buffered saline. Finally, cell apoptosis was detected by flow cytometry (BD Bioscience, Franklin Lakes, NJ, USA) after incubation with annexin V-FITC and propidium iodide for 15 min. Data were acquired with a BD FACSVerse system and BD FACSuite software. (San Jose, CA, USA)

### RNA pull-down

Biotin-labeled, full-length *APOC1P1-3* RNA and antisense *APOC1P1-3* were prepared with Biotin RNA Labeling Mix (Roche, Indianapolis, IN, USA) and T7 RNA polymerase (Roche). Biotinylated RNAs were treated with RNase-free DNase I (Roche) and purified with the RNeasy Mini kit (Qiagen, Valencia, CA, USA). Cell proteins were extracted with the ProteoJETTM Cytoplasmic and Nuclear Protein Extraction kit (Fermentas, St. Leon-Rot, Germany), and then mixed with biotin-labeled RNAs. Washed streptavidin agarose beads (Invitrogen) were added to each binding reaction, incubated at room temperature for 1 h, washed five times and boiled in SDS buffer. Retrieved protein was detected by SDS gel electrophoresis.

### RNA immunoprecipitation

The RIP test was performed with the Magna RIP RNA-Binding Protein Immunoprecipitation kit (Millipore, Bedford, MA, USA) and *α*-tubulin (cat.# 2144, Cell Signaling Technology, Beverly, MA, USA) according to manufacturer's instructions. In brief, beads were mixed with tubulin antibody or IgG and cell lysate, and rotated at room temperature for 4 h. The co-precipitated RNAs were detected by RT-PCR. Total RNAs (input controls) and isotype controls were assayed simultaneously to demonstrate that detected signals were from RNAs, specifically bound to *α*-tubulin.

### Statistical analysis

Data were analyzed using SPSS 17.0 (Chicago, IL, USA). For comparisons, one-way analyses of variance, Fisher's exact tests, *χ*^2^-tests, and two-tailed student's *t*-tests were performed. *P*<0.05 was considered to be statistically significant. The diagrams were completed with Prism 5.0 (GraphPad Software, La Jolla, CA, USA).

## Figures and Tables

**Table 1:** Relationship between *APOC1P1-3* expressions in cancer tissues and clinicopathologic parameters (*n*=90)

	***n***	***APOC1P1-3* expression**^a^		***P*-value**
		**High**	**Low**	
*Menopause*				0.8324
No	41	21	20	
Yes	49	24	25	

*Tumor size* (*cm*)				0.0142^b^
⩾2.5	22	16	6	
<2.5	68	29	39	

*Distant metastasis*				0.5023
Yes	10	6	4	
No	80	39	41	

*Lymph node metastasis*				0.2049
Yes	42	24	18	
No	48	21	27	

*Nottingham grade*				0.5923
Grade I	24	12	12	
Grade II	42	23	19	
Grade III	24	10	14	

*St Gallen subtype*				0.5257
Luminal A	29	17	12	
Luminal B	29	12	17	
HER2 positive	18	8	10	
Basal like	14	8	6	

*ER*				0.8269
⩾1%	57	28	29	
<1%	33	17	16	

*PgR*				0.8324
⩾1%	49	24	25	
<1%	41	21	20	

HER2^c^				0.5165
Overexpression	35	19	16	
Negative	55	26	29	

*ki67*				0.3404
⩾20%	66	35	31	
<20%	24	10	14	

a The cutoff value is the median expression level of *APOC1P1-3* (the value ⩾0.1856 is considered high expression)

b The *APOC1P1-3* expression is related to the tumor size

c HER2 status was validated by fluorescence *in situ* hybridization

## Abbreviations

lncRNA: long non-coding RNA
lincRNA: long intergenic non-coding RNA
APOC1P1: Apolipoprotein C-I pseudogene 1
APOC1: Apolipoprotein C-1
RIP: RNA immunoprecipitation
ER: estrogen receptor
PgR: progesterone receptor
EGFR: epithelial growth factor receptor
