# Supplementary material for: Long intergenic non-coding RNA APOC1P1-3 inhibits apoptosis by decreasing α-tubulin acetylation in breast cancer
Source: Cell Death Dis. 2016 May 26;7(5):e2236–. doi: 10.1038/cddis.2016.142 (PMC4917671; doi:10.1038/cddis.2016.142)
Supplement: Supplementary Information [file cddis2016142x1.docx]

**Supplementary materials and methods**

***Tumor tissues and cell lines***

All fresh breast cancer tissues and matched normal tissues were collected from Shanghai Huashan Hospital, Fudan University. The hematoxylin-eosin stained frozen sections were made firstly to make sure the selected tissue contained enough epithelial elements (more than 70%). Local or systemic treatment had not been conducted prior to the operation. The study was approved by the Research Ethics Committee of Shanghai Medical College, Fudan University. Human breast cancer cell lines BT549, MCF7, MDA-MB-231, MDA-MB-453, MDA-MB-468, SKBR3 and T47D were obtained from Shanghai Type Culture Collection of the Chinese Academy of Sciences, and human mammary epithelial cell line MCF10A were purchased from ATCC. BT549 and T47D were cultured in RPMI-1640 supplemented with 10% fetal bovine serum (FBS) (Gibco, Grand Island, NY USA). MDA-MB-231 and MDA-MB-468 were cultured in L-15 (Gibco) supplemented with 10% FBS without CO_2_. MCF7, MDA-MB-453 and SKBR3 cells were maintained in DMEM (high glucose) (Gibco) supplemented with 10% FBS. MCF10A cell line was maintained in Ham's F12 (Gibco), supplemented with EGF (100 mg/mL) (Sigma, St Louis, MO USA), choleric toxin (100 ng/mL) (Sigma), insulin (10 mg/mL) (Sigma), hydrocortisone (1 mg/mL) (Sigma), 5% horse serum (Gibco).

The clinicopathologic features of the breast cancers selected for lncRNA expression microarray were described in Table S1.

***Real-time quantitative PCR (qPCR)***

Total RNA for cell lines was extracted using TRIzol (Ambion, Carlsbad, CA USA) according to the manufacturer’s instructions. Total RNA for FFPE tissue sections was extracted using the RecoverAll^TM^ Total Nucleic Acid Isolation Kit (Ambion, Austin, Texas USA; containing DNase) according to the manufactures instruction. The last step of the extraction procedure was DNA elimination using DNase incubation for 30 min. RNA was reversed to cDNA using PrimeScript® RT Master Mix at 37 °C for 15 min, 85 °C for 5 sec, and then at 4 °C (for cell lines and fresh tissues, *APOC1P1-3* primers: forward, 5’- AAGGATTCAGGTTGGTGCC -3’, reverse, 5’- TTGGAGACTTCTGGAGCCC -3’; GAPDH primers: forward, 5’- CTGACTTCAACAGCGACACC-3’, reverse, 5’- TGCTGTAGCCAAATTCGTTGT-3’; for paraffin embedded tissues, three primers for *APOC1P1-3*: primer 1: forward, 5’- AAGGATTCAGGTTGGTGCC-3’, reverse, 5’-CGACAGGAAGAGCCTCATG-3’; primer 2: forward, 5’- AGGATTCAGGTTGGTGCCC-3’, reverse, 5’- ACAGAACCACCACCAGGAC-3’; primer 3: forward, 5’- AGGATTCAGGTTGGTGCCC-3’, reverse, 5’-TCGACAGAACCACCACCAG-3’; three primers for GAPDH: primer 1: forward, 5’- TCAAGGCTGAGAACGGGAAG-3’, reverse, 5’- TCCTGGAAGATGGTGATGGG-3’; primer 2: forward, 5’- CCTTCATTGACCTCAACTAC-3’, reverse, 5’- GGGTGGAATCATATTGGAAC-3’; primer 3: forward, 5’- GATTTGGTCGTATTGGGCGC-3’, reverse, 5’- ACCAGAGTTAAAAGCAGCCC-3’; Synthesized by Sangon Biotech, Shanghai, China). qPCR was performed in a 10 μL reaction volume using the SYBR®PremixEx TaqTM and ABI7900HT Real-Time PCR instrument System (Life, Singapore). The thermal cycle condition was that one cycle at 95 °C for 30 sec, followed by 40 cycles of amplification at 95 °C for 5 sec, and then 60 °C for 30 sec. The expression level of objective RNA was normalized to the geometric mean of the conserved gene GAPDH mRNA to control the variability in expression levels and obtained using the calculation as 2^-^^ΔCt^[ΔCt = Ct (*APOC1P1-3*) − Ct (*GAPDH*)], where Ct value represented the threshold cycle for each transcript (for paraffin tissues, the Ct value of is the mean value of the results for three different primers).

***Western blot***

The cells were lysed in ice-cold radioimmunoprecipitation assay buffer supplemented with protease inhibitor and phosphatase inhibitor. Equal amounts of proteins were separated by sodium dodecyl sulfate - polyacrylamide gel electrophoresis (SDS-PAGE) and electrophoretically transferred to polyvinylidene fluoride membranes (Merck Millipore, Darmstadt, Germany). The membranes were blocked with 5% milk in Tris-HCl buffered solution for 1 h, and then incubated with primary antibodies at 4°C overnight and secondary antibodies at 37°C for 1 h. To confirm equal protein loading, the membranes were incubated with actin (Cat.# sc-47778, Santa Cruz, Dallas TX USA) as internal controls. The signals were visualized using an enhanced chemiluminescent substrate and detected by a FluorChem Q imaging system (ProteinSimple, Santa Clara, CA USA). We performed western blot for ER (Cat.# 8644, Cell Signaling, Beverly, MA USA), PgR (Cat.# 8757, Cell Signaling), HER2 (Cat.# sc-33684, Santa Cruz), EGFR (Cat.# 4267, Cell Signaling), Caspase-3 (Cat. #9665, Cell Signaling), α/β-tubulin (Cat. # 2148, Cell Signaling), α-tubulin (Cat. # T6074, Sigma), β-tubulin (Cat.#T2200, Sigma) and Acetyl-α-tubulin (Cat. # 5335, Cell signaling) in this study. Images from western blots were quantified using Quantity One® software (Bio-Rad, Hercules, CA USA). The expression level was normalized with respect to internal reference (β-actin or α-tubulin).

***Immunohistochemistry***

The immunohistochemistry study was performed using Leica BOND-MAX™ fully automated immunohistochemistry system according to the manufacture’s protocol (Leica Microsystems GmbH, Wetzlar, Germany). Briefly, 4-μm-thick sections were deparaffinized and pre-treated with the Epitope Retrieval Solution 2 (EDTA-buffer pH8.8) at 98°C for 20 min. After washing steps, peroxidase blocking was carried out for 10 min using the Bond Polymer Refine Detection Kit DC9800 (Leica Microsystems GmbH). Tissues were again washed and then incubated with the primary antibodies for 60 min. Subsequently, tissues were incubated with polymer for 10 min and developed with 3,3-diaminobenzidine (DAB) for 10 min. In this study we carried out immunohistochemistry in breast cancer tissues for ER (primary antibody was purchased from Dako, Cat. # M3643, Glostrup, Denmark), PgR (Cat. # A0098, Dako), HER2(Leica Bond Oracle HER2 IHC System, Cat.# TA9145, Leica, Shanghai, China), and Ki-67(Cat.# M7240, Dako).

***Cell Counting Kit-8 (CCK8) assay for cell proliferation***

The CCK8 (Dojindo, Rockville, MD USA) assay was performed according to the manufacture’s instruction. The plate included blank wells (medium, 0.1% FBS), control wells (cells, medium, 0.1% FBS), experimental wells (transfection regents, cells, L-15, 0.1% FBS). CCK8 assay was performed after transfection for 24 h. Briefly, 10 μL CCK8 was added in each well, and the optical density (OD) value was detected in a microplate reader at 450 nm (MULTISCAN GO-1510, Thermo Scientific, Shanghai, China) after incubating at 37 °C for 2 h. Each experimental condition was assayed in triplicate and all experiments were performed for three times.

***Cell cycle analysis***

After transfection in serum-free culture for 24 h, cells were maintained in serum-containing medium for 24 h. Thereafter, 1×10^6^ cells were harvested and fixed with ice-cold 75% ethanol overnight. After being washed with ice-cold PBS, cells were incubated with the PI in the dark at room temperature for 30 min. Flow cytometry was performed using a BD FACS Calibur cell sorting system, and the cell cycle phase distribution was calculated using the ModFit LT software.
