# Supplementary material for: Long intergenic non-coding RNA APOC1P1-3 inhibits apoptosis by decreasing α-tubulin acetylation in breast cancer
Source: Cell Death Dis. 2016 May 26;7(5):e2236–. doi: 10.1038/cddis.2016.142 (PMC4917671; doi:10.1038/cddis.2016.142)
Supplement: Supplementary Tables [file cddis2016142x2.docx]

***Supplementary tables***

**Table S1.** Clinicopathologic Characteristics of five cases of breast cancer tissues for microarray

|  | No.2271 | No. 2272 | No. 2273 | No.2274 | No. 2277 |
| --- | --- | --- | --- | --- | --- |
| Age/sex | 45/female | 51/female | 41/female | 61/female | 54/female |
| Tumor size | 3.5 cm | 4.5cm | 4 cm | 3.5cm | 3 cm |
| Pathology | NST* | NST | NST | NST | NST |
| ER | - | - | - | + | + |
| PgR | - | - | - | + (<20%) | + (>20%) |
| HER2 | 3+ | 1+ | 3+ | 1+ | 1+ |
| Ki-67 | 50% | 70% | 40% | 50% | 15% |
| Intrinsic subtype | HER2 positive | Triple negative | HER2 positive | Luminal-B like | Luminal-A like |

Listed are characteristics of the 5 breast cancer cases, whose tissues were used for microarray.

*NST, Invasive carcinoma of no special type

**Table S2.** PCR amplification and sequencing primers for pyrosequencing

|  | **Sequence (5’-3’)** | **5’ labeling** |
| --- | --- | --- |
| APOC1P1-3/1-forward | GTATTAGAGGGTGAATAAGAGTAGATAGAG |  |
| APOC1P1-3/1-reverse | ACAAAACTCTAAATATATACCCCTTCTTCC | Biotin |
| APOC1P1-3/1-sequencing | AAAGGGGGTGAGGTT |  |
| APOC1P1-3/2-forward | TTATTTTGTTTTATTAGGTTGGAGTGTAG |  |
| APOC1P1-3/2-reverse | AACAATATAAAAAAACCTCATCTCTAC | Biotin |
| APOC1P1-3/2-sequencing | GTTGGAGTGTAGTGG |  |
| APOC1P1-3/3-forward | TTGGAGTGTAGTGGAGTAATTATAGTTTAT |  |
| APOC1P1-3/3-reverse | AACAATATAAAAAAACCTCATCTCTAC | Biotin |
| APOC1P1-3/3-sequencing | GAATAGTTGGGATTATAGG |  |
| APOC1P1-3/4-forward | TTTTTGTAGAGATGAGGTTTTTTTATATTG | Biotin |
| APOC1P1-3/4-reverse | CCTACATTAACCAAACATAATAACTCATAC |  |
| APOC1P1-3/4-sequencing | ACATAATAACTCATACCTATAATC |  |
| APOC1P1-3/5-forward | TGAGTTATTATGTTTGGTTAATGTAGGT |  |
| APOC1P1-3/5-reverse | ACTTAATAATTTTCAATCTCCCTTTTTTAC | Biotin |
| APOC1P1-3/5-sequencing | GGTGAGGTTTTTAGTGTT |  |
| APOC1P1-3/6-forward | GGATGTTTTAAGGGTTGGTTTGAAAGAA |  |
| APOC1P1-3/6-reverse | TATAATCTCATCCCCCCCTCATTC | Biotin |
| APOC1P1-3/6-sequencing | TGGTTTGAAAGAAATTGGA |  |
| APOC1P1-3/7-forward | AGGGGAAGTGAGGGAAAGAG |  |
| APOC1P1-3/7-reverse | CTCATCCCCCCCTCATTCA | Biotin |
| APOC1P1-3/7-sequencing | GGGAAAGAGGTGATTTAG |  |
| APOC1P1-3/8-forward | GTGAGGGAAAGAGGTGATTTAG |  |
| APOC1P1-3/8-reverse | CCCTTAACTCCCAAACCCTTTACCA | Biotin |
| APOC1P1-3/8-sequencing | AAGATGGTTATAGGGATAG |  |
| APOC1P1-3/9-forward | GGAGGTGAGGGTGTTGAAT |  |
| APOC1P1-3/9-reverse | CTTCTACCCACCCAAATTTTTCT | Biotin |
| APOC1P1-3/9-sequencing | GGGTGTTGAATGGTAAA |  |
| APOC1P1-3/10-forward | AGGGTTTAGGAGTTAAGGGAAATG |  |
| APOC1P1-3/10-reverse | CTTCTACCCACCCAAATTTTTCT | Biotin |
| APOC1P1-3/10-sequencing | AATGGTTAGGATTTTATTTGA |  |
| APOC1P1-3/11-forward | AGGGGTAGAGGAGAAAAATTTG | Biotin |
| APOC1P1-3/11-reverse | AACACTCAACAACCTAAATCCTTACTAA |  |
| APOC1P1-3/11-sequecing | AAAAAACTTAATTAAAAAATCCTAT |  |

The promoter region and the first exon of the NR_028414. The gray shadow indicates the 11 reactions of the pyrosequencing (the order ascending as *APOC1P1*,2,3,4,5,6,7,8,9,10,11), and the red “cg” in the gray shadow shows the detected sites.

gccagccaaagggggtgaggcccggtggaagggaagaaggggcatacactcagagctttgcagctgaaggttttaattttttgagatggggtctcactctgtctcaccaggctggagtgcagtggcgcaatcacagctcactgcagcctcgaactcctgggctcaagcaatcttcctacctcagcctcttgaatagctgggactacaggtgtgcgccaccacgctcagctaatttttgaacttttttgtagagatgaggtctccctatattgcccaggctggtctcttaactcctgggctcaagtgatcctccttcctcagcttcccaaagcgctgggattacaggcatgagccaccatgcctggccaatgcaggtgaggtttttagtgtccagctaaggcgaccccttccctttgcaaaaaagggagactgaaaatcatcaagttaagagcccagagaatatcagggtggtctgggatgtttcaagggctggtctgaaagaaattggaggtggcacgcagggcagggttgcggggccaactgggaggccccagcaacataaaggaaaagttgttggggctgaggaggcttgctgagagaggggaagtgagggaaagaggtgatctagggacacggtgtgaatgagggggggatgagatcacagggttattactgggagacccctgagggaagatggccacagggacaggacgaggctgtcctctgagtggggaaaggagctatggtagtctgaggaccccccagagtcagggagattgggaggtgagggtgctgaatggtaaagggcttcggagctaagggaaatggtcaggaccccacctgaccccaa

cgcccacgggccaggggcagaggagaaaaacctgggtgggcagaaggaggcaatcttccaggggaaggctcaggaggagggagatcaacatcaacctgccccgccccctccccagcctgataaaggtcctgcg ggcaggacaggacctcc

**Table S3: The small interfering RNA sequence**

| **Name** | **Sense（5'-3'）** | **antisense（5'-3'）** |
| --- | --- | --- |
| siRNA/NC | UUCUCCGAACGUGUCACGUTT | ACGUGACACGUUCGGAGAATT |
| SiRNA/APOC1P1-3 | GGGAAUUCAUCAACCGCAUTT | AUGCGGUUGAUGAAUUCCCTT |

**Table S4.** Differentially expressed lncRNAs screening result using lncRNA microarray (GSE80266)

| **Sequence name** | **FCAbsolute** | ***P*-value** | **Relationship** | **RNAlength** | **Regulation** |
| --- | --- | --- | --- | --- | --- |
| ENST00000441053 | 7.8687596 | 0.010012792 | intergenic | 812 | up |
| G36769 | 7.365211 | 0.038840532 | intergenic | 432 | up |
| ENST00000433123 | 6.036576 | 0.021465804 | intergenic | 1141 | up |
| ENST00000419353 | 5.6566896 | 0.010455364 | intronic antisense | 458 | up |
| ENST00000419353 | 5.6566896 | 0.010455364 | intronic antisense | 458 | up |
| ENST00000429730 | 5.650934 | 0.011012572 | intergenic | 492 | up |
| NR_002712 | 5.606736 | 0.021288132 | intergenic | 2153 | up |
| BF509048 | 5.332398 | 0.04436715 | intergenic | 740 | up |
| ENST00000435078 | 5.2219 | 0.027597671 | intergenic | 939 | up |
| AK096082 | 4.6954556 | 0.016704462 | natural antisense | 1981 | up |
| ENST00000422833 | 4.6755476 | 0.01234163 | intergenic | 115 | up |
| ENST00000438659 | 4.661224 | 0.009172707 | intergenic | 613 | up |
| ENST00000420389 | 4.5392113 | 0.03861305 | intergenic | 539 | up |
| ENST00000457191 | 4.5243154 | 0.020308767 | intergenic | 2729 | up |
| chr2:91595323-91623173+ | 4.485359 | 0.026404925 | intergenic | 27851 | up |
| ENST00000502715 | 4.467742 | 0.001152967 | intronic antisense | 450 | up |
| AY927590 | 4.3488503 | 0.03211832 | intronic antisense | 757 | up |
| ENST00000392566 | 4.296028 | 0.003190995 | intergenic | 462 | up |
| chr6:13349175-13352025- | 3.9639492 | 0.043357708 | intergenic | 2851 | up |
| ENST00000443593 | 3.8040004 | 0.02685357 | intergenic | 1746 | up |
| AA136769 | 3.786105 | 0.029421072 | intergenic | 356 | up |
| ENST00000422732 | 3.673539 | 0.041887067 | intergenic | 2444 | up |
| NR_028412 | 3.5314555 | 0.016717983 | intergenic | 939 | up |
| nc-HOXB4-176- | 3.4989948 | 0.04344614 | intergenic | 158 | up |
| AY605064 | 3.4816756 | 0.024267595 | exon sense-overlapping | 243 | up |
| AY605064 | 3.4816756 | 0.024267595 | exon sense-overlapping | 243 | up |
| ENST00000415020 | 3.4477303 | 0.041611318 | intergenic | 547 | up |
| uc002tft.1 | 3.3872147 | 0.025676586 | intergenic | 3021 | up |
| ENST00000453854 | 3.3138964 | 0.03553204 | intergenic | 435 | up |
| ENST00000506703 | 3.177906 | 0.027519103 | intronic antisense | 552 | up |
| ENST00000506703 | 3.177906 | 0.027519103 | intronic antisense | 552 | up |
| ENST00000506703 | 3.177906 | 0.027519103 | intronic antisense | 552 | up |
| ENST00000506703 | 3.177906 | 0.027519103 | intronic antisense | 552 | up |
| BM699739 | 3.1403568 | 0.013949413 | intergenic | 246 | up |
| HIT000288989 | 3.0312595 | 0.030603305 | natural antisense | 573 | up |
| NR_001587 | 3.0196161 | 0.020353613 | intron sense-overlapping | 1537 | up |
| ENST00000449661 | 2.9660158 | 0.000344307 | intergenic | 238 | up |
| NR_001458 | 2.944311 | 0.032922547 | intergenic | 1500 | up |
| ENST00000456605 | 2.8753963 | 0.006597881 | intergenic | 1438 | up |
| ENST00000414686 | 2.8657577 | 0.006124921 | intronic antisense | 671 | up |
| ENST00000414686 | 2.8657577 | 0.006124921 | intronic antisense | 671 | up |
| ENST00000414686 | 2.8657577 | 0.006124921 | intronic antisense | 671 | up |
| uc003wsp.1 | 2.799221 | 0.029636867 | intergenic | 710 | up |
| AK302893 | 2.6829283 | 0.033124074 | exon sense-overlapping | 3585 | up |
| ENST00000440986 | 2.6743486 | 0.014837937 | intergenic | 684 | up |
| ENST00000515691 | 2.6708982 | 0.049723834 | intergenic | 541 | up |
| uc002kmd.1 | 2.6699944 | 0.021801181 | intergenic | 323 | up |
| ENST00000443304 | 2.638525 | 0.027639821 | intergenic | 400 | up |
| ENST00000430203 | 2.6161675 | 0.023181776 | intronic antisense | 495 | up |
| ENST00000430203 | 2.6161675 | 0.023181776 | intronic antisense | 495 | up |
| AJ844623 | 2.570868 | 0.047675323 | natural antisense | 247 | up |
| ENST00000451536 | 2.478438 | 0.031684175 | intergenic | 493 | up |
| ENST00000426374 | 2.459805 | 0.030538773 | intergenic | 387 | up |
| AK023660 | 2.385645 | 0.027660778 | intergenic | 2046 | up |
| CR613601 | 2.354879 | 0.026796056 | natural antisense | 983 | up |
| NR_026758 | 2.3375103 | 0.03460533 | intergenic | 4525 | up |
| ENST00000433189 | 2.3236804 | 0.024548158 | intergenic | 144 | up |
| ENST00000425609 | 2.3198862 | 0.04893646 | intergenic | 547 | up |
| ENST00000432948 | 2.293092 | 0.04616279 | intergenic | 411 | up |
| chr7:143147778-143169303- | 2.2894084 | 0.049764153 | intergenic | 21526 | up |
| NR_002165 | 2.2740142 | 0.025841909 | intergenic | 888 | up |
| ENST00000417997 | 2.2352846 | 0.009254151 | intergenic | 600 | up |
| NR_027767 | 2.229542 | 0.022570236 | exon sense-overlapping | 768 | up |
| NR_027767 | 2.229542 | 0.022570236 | exon sense-overlapping | 768 | up |
| NR_027767 | 2.229542 | 0.022570236 | exon sense-overlapping | 768 | up |
| NR_027767 | 2.229542 | 0.022570236 | exon sense-overlapping | 768 | up |
| NR_027767 | 2.229542 | 0.022570236 | exon sense-overlapping | 768 | up |
| NR_027767 | 2.229542 | 0.022570236 | exon sense-overlapping | 768 | up |
| NR_027767 | 2.229542 | 0.022570236 | exon sense-overlapping | 768 | up |
| NR_027767 | 2.229542 | 0.022570236 | exon sense-overlapping | 768 | up |
| ENST00000411913 | 2.2272897 | 0.014630933 | intergenic | 584 | up |
| uc003dro.1 | 2.2125683 | 0.019256063 | intergenic | 2595 | up |
| AK024586 | 2.1760702 | 0.038863435 | exon sense-overlapping | 1567 | up |
| NR_002734 | 2.1486604 | 0.009846531 | intronic antisense | 609 | up |
| NR_002734 | 2.1486604 | 0.009846531 | intronic antisense | 609 | up |
| BC047034 | 2.1353054 | 0.02705266 | natural antisense | 1132 | up |
| uc010ijm.1 | 2.1274843 | 0.03663807 | intergenic | 3134 | up |
| ENST00000398012 | 2.1232035 | 0.015805902 | intronic antisense | 1002 | up |
| ENST00000413333 | 2.105299 | 0.043402974 | intergenic | 595 | up |
| ENST00000503768 | 2.053397 | 0.04239808 | intergenic | 1941 | up |
| ENST00000434906 | 2.0525074 | 0.047955513 | intergenic | 739 | up |
| ENST00000515084 | 2.0511286 | 0.0203185 | intergenic | 1972 | up |
| NR_028414 | 2.019401 | 0.017554004 | intergenic | 631 | up |
| uc003qir.2 | 1.9765116 | 0.01307968 | intergenic | 1292 | up |
| BC038776 | 1.9760654 | 0.027573809 | bidirectional | 915 | up |
| BC038776 | 1.9760654 | 0.027573809 | bidirectional | 915 | up |
| ENST00000450749 | 1.9745828 | 1.99617E-05 | intergenic | 678 | up |
| ENST00000423135 | 1.973034 | 0.005738572 | intergenic | 221 | up |
| ENST00000378248 | 1.9541727 | 0.006415822 | intronic antisense | 2924 | up |
| BC015321 | 1.9505752 | 0.013996472 | natural antisense | 1792 | up |
| ENST00000429872 | 1.9473505 | 0.03754951 | intronic antisense | 731 | up |
| ENST00000429872 | 1.9473505 | 0.03754951 | intronic antisense | 731 | up |
| ENST00000429872 | 1.9473505 | 0.03754951 | intronic antisense | 731 | up |
| ENST00000429872 | 1.9473505 | 0.03754951 | intronic antisense | 731 | up |
| ENST00000429872 | 1.9473505 | 0.03754951 | intronic antisense | 731 | up |
| ENST00000429872 | 1.9473505 | 0.03754951 | intronic antisense | 731 | up |
| ENST00000508435 | 1.9464123 | 0.013053438 | intergenic | 827 | up |
| ENST00000508037 | 1.9431244 | 0.024834152 | intergenic | 287 | up |
| NR_024373 | 1.9399447 | 0.005211397 | intergenic | 557 | up |
| HIT000320855 | 1.939032 | 0.022229493 | natural antisense | 528 | up |
| ENST00000363006 | 1.929195 | 0.03567851 | intergenic | 301 | up |
| ENST00000436148 | 1.9262781 | 0.04474322 | intergenic | 475 | up |
| ENST00000439562 | 1.9227492 | 0.049931392 | natural antisense | 694 | up |
| ENST00000439562 | 1.9227492 | 0.049931392 | natural antisense | 694 | up |
| ENST00000439562 | 1.9227492 | 0.049931392 | natural antisense | 694 | up |
| ENST00000443015 | 1.9185429 | 0.0268151 | intronic antisense | 339 | up |
| ENST00000443015 | 1.9185429 | 0.0268151 | intronic antisense | 339 | up |
| ENST00000483806 | 1.9146768 | 0.011333411 | intergenic | 638 | up |
| NR_026583 | 1.9146737 | 0.006675264 | intergenic | 2794 | up |
| ENST00000392141 | 1.8975439 | 0.03340728 | intergenic | 600 | up |
| ENST00000419546 | 1.8874059 | 0.012771975 | intergenic | 1489 | up |
| AK022198 | 1.8806571 | 0.042976793 | intergenic | 1905 | up |
| ENST00000446748 | 1.8716457 | 0.036922153 | intergenic | 202 | up |
| uc002knb.2 | 1.8679074 | 0.027035324 | intronic antisense | 2044 | up |
| ENST00000443631 | 1.8371016 | 0.031677764 | bidirectional | 544 | up |
| ENST00000443631 | 1.8371016 | 0.031677764 | intronic antisense | 544 | up |
| BC047582 | 1.8328379 | 0.04701192 | bidirectional | 2283 | up |
| NR_024444 | 1.8295678 | 0.025248915 | intergenic | 661 | up |
| ENST00000434244 | 1.8282404 | 0.040092416 | intergenic | 2644 | up |
| ENST00000449709 | 1.8206869 | 0.03600891 | intronic antisense | 577 | up |
| NR_002454 | 1.8185616 | 0.011749602 |  | 571 | up |
| ENST00000440334 | 1.8077135 | 0.033756852 | intergenic | 420 | up |
| ENST00000406327 | 1.8010412 | 0.013973801 | intergenic | 513 | up |
| ENST00000400805 | 1.7864627 | 0.034865726 | intergenic | 616 | up |
| ENST00000431512 | 1.7818195 | 0.04365204 | natural antisense | 394 | up |
| ENST00000405405 | 1.7734318 | 0.03805512 | intergenic | 1007 | up |
| uc003wmd.1 | 1.7700809 | 0.005388885 | bidirectional | 2946 | up |
| ENST00000512896 | 1.7662449 | 0.016364917 | intronic antisense | 1047 | up |
| ENST00000415070 | 1.7496045 | 0.009627314 | intronic antisense | 132 | up |
| ENST00000415070 | 1.7496045 | 0.009627314 | intronic antisense | 132 | up |
| BC047792 | 1.7491789 | 0.020308092 | natural antisense | 1475 | up |
| uc001esd.2 | 1.7471126 | 0.001502889 | natural antisense | 729 | up |
| uc001esd.2 | 1.7471126 | 0.001502889 | intronic antisense | 729 | up |
| AF143741 | 1.741213 | 0.025361218 | natural antisense | 694 | up |
| ENST00000451485 | 1.7387022 | 0.025098957 | intronic antisense | 656 | up |
| ENST00000451485 | 1.7387022 | 0.025098957 | bidirectional | 656 | up |
| ENST00000451485 | 1.7387022 | 0.025098957 | bidirectional | 656 | up |
| ENST00000451485 | 1.7387022 | 0.025098957 | intronic antisense | 656 | up |
| AK095096 | 1.7352254 | 0.027466254 | intergenic | 3662 | up |
| ENST00000440946 | 1.7294568 | 0.04280431 | intergenic | 1160 | up |
| CR622106 | 1.7230488 | 0.03505922 | intergenic | 1617 | up |
| CF594357 | 1.7115589 | 0.026341127 | intergenic | 801 | up |
| uc002efy.2 | 1.7113129 | 0.017142177 | intergenic | 544 | up |
| uc001elf.3 | 1.7087277 | 0.005645054 | intergenic | 704 | up |
| AK024123 | 1.7078458 | 0.04661566 | intron sense-overlapping | 2336 | up |
| AK024123 | 1.7078458 | 0.04661566 | intron sense-overlapping | 2336 | up |
| ENST00000428364 | 1.7059784 | 0.048958797 | intergenic | 672 | up |
| AX721245 | 1.7026931 | 0.041403174 | intronic antisense | 455 | up |
| AX721245 | 1.7026931 | 0.041403174 | intronic antisense | 455 | up |
| AX721245 | 1.7026931 | 0.041403174 | intronic antisense | 455 | up |
| BC035326 | 1.7022272 | 0.024344157 | intronic antisense | 1835 | up |
| AL050074 | 1.697634 | 0.020047627 | exon sense-overlapping | 2221 | up |
| AF086374 | 1.6973435 | 0.030463489 | intergenic | 707 | up |
| AK057050 | 1.6920687 | 0.03183758 | intergenic | 1971 | up |
| BC030956 | 1.6856092 | 0.01779807 | exon sense-overlapping | 1970 | up |
| BC030956 | 1.6856092 | 0.01779807 | natural antisense | 1970 | up |
| uc010ixi.1 | 1.6823487 | 0.023169603 | intergenic | 1073 | up |
| ENST00000445829 | 1.6804392 | 0.008898125 | intergenic | 389 | up |
| ENST00000425725 | 1.6721779 | 0.00305937 | intergenic | 597 | up |
| ENST00000413035 | 1.6688987 | 0.028443886 | natural antisense | 839 | up |
| ENST00000413035 | 1.6688987 | 0.028443886 | natural antisense | 839 | up |
| ENST00000413035 | 1.6688987 | 0.028443886 | natural antisense | 839 | up |
| BC012429 | 1.6634319 | 0.007962906 | intergenic | 1396 | up |
| NR_024528 | 1.6622931 | 0.007152364 | bidirectional | 1482 | up |
| NR_024528 | 1.6622931 | 0.007152364 | bidirectional | 1482 | up |
| D55639 | 1.6584456 | 0.014930748 | natural antisense | 1323 | up |
| D55639 | 1.6584456 | 0.014930748 | natural antisense | 1323 | up |
| ENST00000427935 | 1.6569514 | 0.043870118 | intergenic | 228 | up |
| ENST00000454989 | 1.6540738 | 0.005224728 | intergenic | 520 | up |
| nc-HOXA13-96- | 1.6502624 | 0.001263944 | intergenic | 318 | up |
| ENST00000439127 | 1.6343169 | 0.008713861 | intergenic | 1412 | up |
| ENST00000514188 | 1.6342013 | 0.014777588 | intergenic | 555 | up |
| ENST00000424793 | 1.6337914 | 0.044768754 | intergenic | 1781 | up |
| ENST00000432117 | 1.6193646 | 0.007702029 | intergenic | 741 | up |
| uc001xyn.2 | 1.6154269 | 0.010063113 | intergenic | 1375 | up |
| ENST00000424182 | 1.6148142 | 0.03135619 | intergenic | 230 | up |
| ENST00000453692 | 1.6146386 | 0.048125952 | natural antisense | 758 | up |
| ENST00000482834 | 1.6128395 | 0.017579732 | intergenic | 794 | up |
| NR_030697 | 1.612443 | 0.014782471 | exon sense-overlapping | 706 | up |
| NR_030697 | 1.612443 | 0.014782471 | bidirectional | 706 | up |
| NR_030697 | 1.612443 | 0.014782471 | exon sense-overlapping | 706 | up |
| NR_030697 | 1.612443 | 0.014782471 | bidirectional | 706 | up |
| ENST00000447119 | 1.608875 | 0.040117938 | intronic antisense | 613 | up |
| uc003yed.2 | 1.6083397 | 0.04944156 | bidirectional | 1430 | up |
| ENST00000502532 | 1.6020724 | 0.04359459 | intergenic | 2685 | up |
| NR_024174 | 1.6003492 | 0.023866931 | exon sense-overlapping | 2575 | up |
| NR_024174 | 1.6003492 | 0.023866931 | exon sense-overlapping | 2575 | up |
| NR_024174 | 1.6003492 | 0.023866931 | exon sense-overlapping | 2575 | up |
| ENST00000435106 | 1.599077 | 0.040841747 | intergenic | 1251 | up |
| ENST00000318333 | 1.5958848 | 0.03827532 | intronic antisense | 959 | up |
| ENST00000507312 | 1.5925046 | 0.004353653 | intergenic | 1168 | up |
| ENST00000516854 | 1.5859084 | 0.033708114 | intergenic | 280 | up |
| ENST00000506718 | 1.5802412 | 0.040917106 | intergenic | 2762 | up |
| ENST00000394213 | 1.5786532 | 0.015124238 | intronic antisense | 387 | up |
| ENST00000394213 | 1.5786532 | 0.015124238 | intronic antisense | 387 | up |
| ENST00000483867 | 1.572686 | 0.002894221 | intergenic | 983 | up |
| ENST00000502317 | 1.5691128 | 0.036245674 | intergenic | 768 | up |
| AL157421 | 1.5662309 | 0.03861749 | natural antisense | 1698 | up |
| ENST00000511867 | 1.5615953 | 0.037798513 | intergenic | 586 | up |
| ENST00000418454 | 1.5595368 | 0.044142507 | intergenic | 1082 | up |
| NR_003598 | 1.5487686 | 0.027233854 | intergenic | 1332 | up |
| ENST00000453682 | 1.5461617 | 0.013112783 | intergenic | 566 | up |
| ENST00000231383 | 1.540216 | 0.007275449 | intergenic | 813 | up |
| NR_033184 | 1.534243 | 0.008236824 | exon sense-overlapping | 8593 | up |
| ENST00000460239 | 1.5283154 | 0.028709084 | intergenic | 581 | up |
| chr2:121182275-121194039+ | 1.5268543 | 0.03792817 | intergenic | 11765 | up |
| AL390159 | 1.5265732 | 0.008644102 | intergenic | 4158 | up |
| CR595946 | 1.5256032 | 0.006130394 | exon sense-overlapping | 660 | up |
| CR595946 | 1.5256032 | 0.006130394 | exon sense-overlapping | 660 | up |
| CR595946 | 1.5256032 | 0.006130394 | exon sense-overlapping | 660 | up |
| CR595946 | 1.5256032 | 0.006130394 | exon sense-overlapping | 660 | up |
| CR595946 | 1.5256032 | 0.006130394 | exon sense-overlapping | 660 | up |
| CR595946 | 1.5256032 | 0.006130394 | exon sense-overlapping | 660 | up |
| ENST00000505368 | 1.5233781 | 0.023357337 | intergenic | 637 | up |
| ENST00000504840 | 1.5186917 | 0.033816565 | intergenic | 702 | up |
| ENST00000420185 | 1.5186485 | 0.039117444 | intergenic | 1589 | up |
| Y16708 | 1.5144005 | 0.008712661 | natural antisense | 272 | up |
| nc-HOXD3-38+ | 1.5052679 | 0.0393426 | intergenic | 208 | up |
| NR_024332 | 1.5043119 | 0.035944622 | exon sense-overlapping | 2953 | up |
| NR_024332 | 1.5043119 | 0.035944622 | natural antisense | 2953 | up |
| ENST00000505635 | 1.5022464 | 0.039320752 | intergenic | 4332 | up |
| uc010crb.1 | 1.5021158 | 0.001116083 | intergenic | 1080 | up |
| ENST00000510482 | 1.5017266 | 0.017424753 | intergenic | 3903 | up |
| ENST00000447194 | 1.5010384 | 0.017373135 | intergenic | 2852 | up |
| ENST00000422010 | 2.0322254 | 0.011196365 | intergenic | 599 | down |
| ENST00000434311 | 1.5086069 | 0.026220955 | intergenic | 478 | down |
| ENST00000499517 | 1.6011523 | 0.045032714 | intergenic | 2944 | down |
| ENST00000366287 | 2.0811985 | 0.0438041 | natural antisense | 771 | down |
| ENST00000366287 | 2.0811985 | 0.0438041 | natural antisense | 771 | down |
| ENST00000423288 | 1.8111684 | 0.000129136 | intergenic | 2910 | down |
| uc001alp.1 | 1.6128032 | 0.04226458 | intergenic | 847 | down |
| chr9:13713659-13719345- | 1.688189 | 0.000868772 | intergenic | 5687 | down |
| ENST00000412238 | 1.621185 | 0.001870409 | intergenic | 351 | down |
| uc001eng.1 | 1.6801294 | 0.010695415 | intergenic | 1711 | down |
| ENST00000449899 | 1.7908771 | 0.024947233 | intergenic | 543 | down |
| ENST00000425419 | 1.7041372 | 0.008397748 | intronic antisense | 552 | down |
| ENST00000425419 | 1.7041372 | 0.008397748 | intronic antisense | 552 | down |
| ENST00000425419 | 1.7041372 | 0.008397748 | intronic antisense | 552 | down |
| uc003zpr.2 | 1.5674168 | 0.03552929 | intergenic | 4804 | down |
| chr2:107718693-107737818+ | 2.1752224 | 0.011176164 | intergenic | 19126 | down |
| ENST00000509304 | 1.6069698 | 0.022154395 | intergenic | 709 | down |
| ENST00000432653 | 2.537919 | 0.000917145 | intronic antisense | 470 | down |
| ENST00000432653 | 2.537919 | 0.000917145 | intronic antisense | 470 | down |
| ENST00000432653 | 2.537919 | 0.000917145 | intronic antisense | 470 | down |
| ENST00000432653 | 2.537919 | 0.000917145 | intronic antisense | 470 | down |
| BC009060 | 1.557882 | 0.007794136 | intergenic | 1247 | down |
| uc.358- | 1.7627248 | 0.022618681 | intergenic | 226 | down |
| ENST00000504655 | 1.6691072 | 0.04509466 | intergenic | 968 | down |
| ENST00000503465 | 1.8865708 | 0.012010652 | intergenic | 500 | down |
| AF116626 | 2.29714 | 9.86238E-05 | intronic antisense | 1326 | down |
| AF116626 | 2.29714 | 9.86238E-05 | intronic antisense | 1326 | down |
| AF116626 | 2.29714 | 9.86238E-05 | intronic antisense | 1326 | down |
| AF116626 | 2.29714 | 9.86238E-05 | intronic antisense | 1326 | down |
| ENST00000503317 | 1.7047203 | 0.012406965 | intergenic | 558 | down |
| ENST00000428512 | 1.576456 | 0.028424822 | intronic antisense | 440 | down |
| ENST00000428512 | 1.576456 | 0.028424822 | intronic antisense | 440 | down |
| ENST00000426456 | 1.7518067 | 0.015294216 | intergenic | 532 | down |
| ENST00000503987 | 1.5128771 | 0.006185754 | intergenic | 763 | down |
| AK127738 | 1.7957665 | 0.013599019 | natural antisense | 2366 | down |
| ENST00000500420 | 1.5549437 | 0.038426697 | intergenic | 1885 | down |
| ENST00000415124 | 1.933362 | 0.043733828 | natural antisense | 352 | down |
| ENST00000415124 | 1.933362 | 0.043733828 | natural antisense | 352 | down |
| ENST00000415124 | 1.933362 | 0.043733828 | natural antisense | 352 | down |
| chr11:44720724-44741649+ | 1.5262089 | 0.025802122 | intergenic | 20926 | down |
| CD048049 | 2.0391204 | 0.023345532 | exon sense-overlapping | 923 | down |
| ENST00000447784 | 1.798028 | 0.006830627 | intergenic | 560 | down |
| ENST00000427717 | 2.1350534 | 0.008060645 | intergenic | 431 | down |
| NR_003015 | 1.8234445 | 0.044257578 | intron sense-overlapping | 250 | down |
| NR_003015 | 1.8234445 | 0.044257578 | intron sense-overlapping | 250 | down |
| NR_003015 | 1.8234445 | 0.044257578 | intron sense-overlapping | 250 | down |
| BX111087 | 2.2533433 | 0.001546695 | intergenic | 701 | down |
| G43240 | 1.56065 | 0.04853834 | natural antisense | 293 | down |
| G43240 | 1.56065 | 0.04853834 | natural antisense | 293 | down |
| G43240 | 1.56065 | 0.04853834 | natural antisense | 293 | down |
| G43240 | 1.56065 | 0.04853834 | intronic antisense | 293 | down |
| NR_027086 | 4.215887 | 0.04777632 | intergenic | 2363 | down |
| ENST00000513620 | 1.6673472 | 0.032203533 | intergenic | 788 | down |
| NR_002946 | 1.5897578 | 0.03067689 | exon sense-overlapping | 906 | down |
| nc-HOXC6-243- | 1.7303401 | 0.005734859 | intron sense-overlapping | 958 | down |
| nc-HOXC6-243- | 1.7303401 | 0.005734859 | intron sense-overlapping | 958 | down |
| chr2:122573205-122584730- | 1.5523078 | 0.002823657 | intergenic | 11526 | down |
| ENST00000507838 | 1.9314715 | 0.007163922 | intergenic | 2163 | down |
| uc001fuu.2 | 1.6746829 | 0.034079906 | intergenic | 423 | down |
| ENST00000454965 | 1.7467223 | 0.017639818 | intergenic | 348 | down |
| uc003imx.1 | 1.7902621 | 0.04245685 | intronic antisense | 2371 | down |
| ENST00000455088 | 1.6983277 | 0.021684317 | intergenic | 2102 | down |
| NR_024569 | 1.5156573 | 0.029193755 | exon sense-overlapping | 5001 | down |
| ENST00000436102 | 1.6194525 | 0.035387475 | intergenic | 764 | down |
| ENST00000366405 | 1.7831355 | 0.002823116 | intergenic | 598 | down |
| NR_029429 | 1.5781826 | 0.001782612 | intergenic | 608 | down |
| AI553950 | 1.9909103 | 0.005307484 | natural antisense | 449 | down |
| G36585 | 1.5417557 | 0.024378804 | intronic antisense | 475 | down |
| G36585 | 1.5417557 | 0.024378804 | intronic antisense | 475 | down |
| G36585 | 1.5417557 | 0.024378804 | intronic antisense | 475 | down |
| G36585 | 1.5417557 | 0.024378804 | intronic antisense | 475 | down |
| uc003mtu.1 | 1.6595544 | 0.022095298 | intergenic | 566 | down |
| ENST00000433121 | 1.5414801 | 0.044609312 | intergenic | 477 | down |
| ENST00000505398 | 1.6771228 | 0.014110112 | intronic antisense | 623 | down |
| ENST00000440556 | 1.6387231 | 0.006747505 | intergenic | 1771 | down |
| DQ656008 | 1.5698141 | 0.03784487 | natural antisense | 1013 | down |
| W19080 | 1.6027731 | 0.019650022 | intergenic | 469 | down |
| ENST00000305671 | 2.0376437 | 0.000824276 | intergenic | 201 | down |
| AK129642 | 2.2402935 | 0.006800244 | intron sense-overlapping | 1618 | down |
| ENST00000511318 | 1.5731424 | 0.01556355 | intergenic | 336 | down |
| BC043263 | 1.6325753 | 0.03716218 | intergenic | 2325 | down |
| ENST00000425176 | 2.0490937 | 0.015492854 | intergenic | 317 | down |
| uc001dfx.2 | 1.6422398 | 0.001459145 | intergenic | 3870 | down |
| ENST00000500980 | 1.5076872 | 0.017032364 | natural antisense | 1913 | down |
| ENST00000500980 | 1.5076872 | 0.017032364 | natural antisense | 1913 | down |
| ENST00000500980 | 1.5076872 | 0.017032364 | natural antisense | 1913 | down |
| ENST00000500980 | 1.5076872 | 0.017032364 | natural antisense | 1913 | down |
| ENST00000428542 | 1.8129946 | 0.006714387 | intergenic | 523 | down |
| ENST00000449338 | 1.5279619 | 0.04948775 | intronic antisense | 276 | down |
| AK124839 | 1.8011429 | 0.035658665 | natural antisense | 2325 | down |
| AW361687 | 1.8302392 | 0.049901456 | intronic antisense | 601 | down |
| AW361687 | 1.8302392 | 0.049901456 | intron sense-overlapping | 601 | down |
| AW361687 | 1.8302392 | 0.049901456 | intronic antisense | 601 | down |
| AW361687 | 1.8302392 | 0.049901456 | intron sense-overlapping | 601 | down |
| AW361687 | 1.8302392 | 0.049901456 | intronic antisense | 601 | down |
| AW361687 | 1.8302392 | 0.049901456 | intronic antisense | 601 | down |
| AW361687 | 1.8302392 | 0.049901456 | intronic antisense | 601 | down |
| AW361687 | 1.8302392 | 0.049901456 | intron sense-overlapping | 601 | down |
| AW361687 | 1.8302392 | 0.049901456 | exon sense-overlapping | 601 | down |
| AW361687 | 1.8302392 | 0.049901456 | intron sense-overlapping | 601 | down |
| ENST00000425193 | 1.5787106 | 0.032521803 | intergenic | 546 | down |
| ENST00000444350 | 1.5750862 | 0.020771379 | intergenic | 727 | down |
| uc002vrh.1 | 1.6849341 | 0.018127663 | bidirectional | 1250 | down |
| ENST00000436538 | 1.5965526 | 0.019336218 | intergenic | 1642 | down |
| ENST00000443744 | 1.7065847 | 0.018746087 | intergenic | 1896 | down |
| ENST00000477499 | 1.5418607 | 0.002113424 | intergenic | 787 | down |
| ENST00000361014 | 1.6752105 | 0.005655975 | intergenic | 1404 | down |
| AY726573 | 1.9051197 | 0.01623895 | intergenic | 771 | down |
| BG003184 | 1.930929 | 0.034463555 | intergenic | 408 | down |
| BC040238 | 2.3390691 | 0.031980928 | intergenic | 3614 | down |
| ENST00000443679 | 1.7152542 | 0.045182526 | intergenic | 285 | down |
| N48415 | 1.6133434 | 0.008559329 | intergenic | 541 | down |
| BC045654 | 1.6482618 | 0.012601436 | intergenic | 1210 | down |
| NR_027139 | 1.5022479 | 0.019849192 | exon sense-overlapping | 1721 | down |
| uc002eyt.2 | 1.7308613 | 0.01889202 | natural antisense | 1470 | down |
| uc002eyt.2 | 1.7308613 | 0.01889202 | natural antisense | 1470 | down |
| uc002eyt.2 | 1.7308613 | 0.01889202 | natural antisense | 1470 | down |
| uc002eyt.2 | 1.7308613 | 0.01889202 | bidirectional | 1470 | down |
| ENST00000507161 | 1.7558969 | 0.032097388 | bidirectional | 2525 | down |
| ENST00000444386 | 1.7097231 | 0.015424938 | natural antisense | 545 | down |
| ENST00000444386 | 1.7097231 | 0.015424938 | natural antisense | 545 | down |
| AF086199 | 1.7115188 | 0.025594858 | intergenic | 457 | down |
| NR_024622 | 1.93609 | 0.027154433 | bidirectional | 1215 | down |
| NR_024622 | 1.93609 | 0.027154433 | bidirectional | 1215 | down |
| NR_024622 | 1.93609 | 0.027154433 | intronic antisense | 1215 | down |
| NR_024622 | 1.93609 | 0.027154433 | intronic antisense | 1215 | down |
| NR_024622 | 1.93609 | 0.027154433 | intronic antisense | 1215 | down |
| ENST00000513608 | 1.6922269 | 0.00868476 | intergenic | 526 | down |
| NR_026780 | 1.722332 | 0.014590044 | intergenic | 2566 | down |
| ENST00000432822 | 1.8093626 | 0.026977472 | intergenic | 443 | down |
| BC019855 | 1.7937406 | 0.009389179 | natural antisense | 1379 | down |
| ENST00000430821 | 1.8041315 | 0.002457551 | intergenic | 400 | down |
| BC050304 | 1.9270359 | 0.024143418 | intergenic | 1798 | down |
| ENST00000457832 | 1.5843701 | 0.01903178 | intergenic | 1222 | down |
| chr9:20285050-20323050+ | 1.719866 | 0.013799788 | intergenic | 38001 | down |
| NR_003668 | 1.8243301 | 0.008634128 | intergenic | 264 | down |
| AY927602 | 1.5652466 | 0.0279784 | exon sense-overlapping | 828 | down |
| AY927602 | 1.5652466 | 0.0279784 | exon sense-overlapping | 828 | down |
| AY927602 | 1.5652466 | 0.0279784 | exon sense-overlapping | 828 | down |
| AY927602 | 1.5652466 | 0.0279784 | exon sense-overlapping | 828 | down |
| AY927602 | 1.5652466 | 0.0279784 | exon sense-overlapping | 828 | down |
| NR_027297 | 1.5248923 | 0.009391488 | exon sense-overlapping | 1991 | down |
| NR_027297 | 1.5248923 | 0.009391488 | exon sense-overlapping | 1991 | down |
| NR_027297 | 1.5248923 | 0.009391488 | exon sense-overlapping | 1991 | down |
| NR_027297 | 1.5248923 | 0.009391488 | exon sense-overlapping | 1991 | down |
| ENST00000502455 | 1.9805654 | 0.025220077 | bidirectional | 549 | down |
| ENST00000502455 | 1.9805654 | 0.025220077 | bidirectional | 549 | down |
| uc002isy.2 | 1.7114425 | 0.010718652 | intergenic | 1179 | down |
| uc002uav.1 | 1.6220415 | 0.019701432 | natural antisense | 1494 | down |
| HIT000191751 | 1.9020618 | 0.006709198 | intergenic | 336 | down |
| chr9:81923716-81932183- | 1.657737 | 0.019327683 | intergenic | 8468 | down |
| HIT000387198 | 1.9036839 | 0.012829495 | natural antisense | 446 | down |
| HIT000387198 | 1.9036839 | 0.012829495 | intronic antisense | 446 | down |
| BX648461 | 1.5616589 | 0.032237872 | natural antisense | 4824 | down |
| ENST00000420237 | 1.7506815 | 0.027653152 | intergenic | 1126 | down |
| ENST00000436848 | 1.7247719 | 0.03397362 | natural antisense | 258 | down |
| ENST00000436848 | 1.7247719 | 0.03397362 | natural antisense | 258 | down |
| ENST00000272546 | 2.6216788 | 0.016379414 | intergenic | 369 | down |
| AI347010 | 1.5025752 | 0.049572952 | natural antisense | 471 | down |
| AI347010 | 1.5025752 | 0.049572952 | natural antisense | 471 | down |
| AI347010 | 1.5025752 | 0.049572952 | intron sense-overlapping | 471 | down |
| AI347010 | 1.5025752 | 0.049572952 | natural antisense | 471 | down |
| uc.129+ | 2.0138168 | 0.020732306 | intron sense-overlapping | 212 | down |
| uc.129+ | 2.0138168 | 0.020732306 | intron sense-overlapping | 212 | down |
| uc.129+ | 2.0138168 | 0.020732306 | exon sense-overlapping | 212 | down |
| uc.129+ | 2.0138168 | 0.020732306 | intron sense-overlapping | 212 | down |
| uc.129+ | 2.0138168 | 0.020732306 | intron sense-overlapping | 212 | down |
| uc.129+ | 2.0138168 | 0.020732306 | intron sense-overlapping | 212 | down |
| uc.129+ | 2.0138168 | 0.020732306 | intron sense-overlapping | 212 | down |
| chr4:158983666-159005030+ | 1.7534362 | 0.025893416 | intergenic | 21365 | down |
| uc003bwp.1 | 2.7468886 | 0.006591713 | intergenic | 657 | down |
| ENST00000430717 | 2.7822363 | 0.02047168 | intergenic | 1041 | down |
| ENST00000435024 | 1.547246 | 0.04294159 | intergenic | 631 | down |
| G36947 | 2.071439 | 0.011579669 | natural antisense | 411 | down |
| G36947 | 2.071439 | 0.011579669 | natural antisense | 411 | down |
| AK130560 | 1.9856012 | 0.010181829 | intergenic | 1499 | down |
| uc.212- | 1.6809837 | 0.005772072 | exon sense-overlapping | 205 | down |
| ENST00000507626 | 1.7123591 | 0.013666625 | intergenic | 1588 | down |
| NR_026789 | 1.7466831 | 0.022866543 | intergenic | 775 | down |
| AF085902 | 1.599381 | 0.013325976 | intronic antisense | 401 | down |
| uc011mle.1 | 1.5878097 | 0.00742508 | intergenic | 110 | down |
| ENST00000443247 | 1.6691017 | 0.002022935 | intergenic | 1135 | down |
| AF161558 | 1.6435523 | 0.042916223 | intergenic | 702 | down |
| NR_023312 | 1.9993451 | 0.012719099 | exon sense-overlapping | 3312 | down |
| HIT000068211 | 1.6461282 | 0.041465104 | natural antisense | 537 | down |
| HIT000068211 | 1.6461282 | 0.041465104 | natural antisense | 537 | down |
| ENST00000391359 | 1.7402418 | 0.015223621 | intergenic | 544 | down |
| ENST00000514358 | 1.5211452 | 0.013888666 | intergenic | 690 | down |
| AY295762 | 1.5528297 | 0.030679645 | natural antisense | 609 | down |
| AY295762 | 1.5528297 | 0.030679645 | natural antisense | 609 | down |
| G43356 | 1.8458947 | 0.010503334 | natural antisense | 150 | down |
| G43356 | 1.8458947 | 0.010503334 | natural antisense | 150 | down |
| G43356 | 1.8458947 | 0.010503334 | natural antisense | 150 | down |
| G43356 | 1.8458947 | 0.010503334 | natural antisense | 150 | down |
| G43356 | 1.8458947 | 0.010503334 | natural antisense | 150 | down |
| G43356 | 1.8458947 | 0.010503334 | natural antisense | 150 | down |
| G43356 | 1.8458947 | 0.010503334 | natural antisense | 150 | down |
| G43356 | 1.8458947 | 0.010503334 | natural antisense | 150 | down |
| uc001ayw.2 | 1.8165718 | 0.014518072 | intergenic | 1160 | down |
| AK299029 | 2.6710784 | 0.033299275 | exon sense-overlapping | 2776 | down |
| AK299029 | 2.6710784 | 0.033299275 | exon sense-overlapping | 2776 | down |
| AK299029 | 2.6710784 | 0.033299275 | exon sense-overlapping | 2776 | down |
| AK094786 | 2.075772 | 0.0399077 | intergenic | 2661 | down |
| ENST00000503118 | 1.6079246 | 0.045016807 | natural antisense | 421 | down |
| ENST00000503118 | 1.6079246 | 0.045016807 | natural antisense | 421 | down |
| ENST00000440633 | 1.5404502 | 0.035816506 | intergenic | 411 | down |
| AF056433 | 1.6155871 | 0.020955293 | intergenic | 1735 | down |
| uc003vql.1 | 1.7078983 | 0.03912244 | intergenic | 1618 | down |
| NR_003013 | 2.2100592 | 0.031528395 | intron sense-overlapping | 187 | down |
| NR_003013 | 2.2100592 | 0.031528395 | intron sense-overlapping | 187 | down |
| NR_002555 | 1.6262301 | 0.03925896 | intergenic | 3455 | down |
| AK123655 | 1.5503747 | 0.037712045 | exon sense-overlapping | 2349 | down |
| AK123655 | 1.5503747 | 0.037712045 | exon sense-overlapping | 2349 | down |
| AK055723 | 1.9511626 | 0.003265023 | intergenic | 2442 | down |
| ENST00000432228 | 1.667123 | 0.047025755 | intergenic | 2029 | down |
| NR_027921 | 1.77827 | 0.005874337 | exon sense-overlapping | 1487 | down |
| NR_027921 | 1.77827 | 0.005874337 | exon sense-overlapping | 1487 | down |
| NR_027921 | 1.77827 | 0.005874337 | exon sense-overlapping | 1487 | down |
| ENST00000444292 | 1.5286695 | 0.031533618 | intergenic | 981 | down |
| ENST00000442557 | 1.6217504 | 0.005513371 | intronic antisense | 355 | down |
| uc002tjx.3 | 3.1618302 | 0.04447231 | intergenic | 536 | down |
| AK123086 | 1.5709809 | 0.043753378 | intronic antisense | 2339 | down |
| HIT000066914 | 1.7356608 | 0.04564551 | intronic antisense | 361 | down |
| ENST00000415467 | 1.529698 | 0.040895168 | bidirectional | 760 | down |
| ENST00000415467 | 1.529698 | 0.040895168 | bidirectional | 760 | down |
| ENST00000415467 | 1.529698 | 0.040895168 | bidirectional | 760 | down |
| ENST00000415467 | 1.529698 | 0.040895168 | bidirectional | 760 | down |
| nc-HOXD1-46- | 1.8925232 | 0.023335814 | intergenic | 2057 | down |
| uc009voq.1 | 1.754427 | 0.002555445 | intergenic | 2482 | down |
| AL080082 | 1.9707249 | 0.042460017 | intergenic | 1026 | down |
| ENST00000499207 | 1.5719008 | 0.04016614 | natural antisense | 1770 | down |
| AK055220 | 1.7771177 | 0.009967875 | intergenic | 2643 | down |
| ENST00000417082 | 1.8894019 | 0.049111094 | intergenic | 1092 | down |
| uc001elr.3 | 1.7094451 | 0.022729527 | intergenic | 1417 | down |
| uc003tvo.2 | 1.5130222 | 0.006151646 | bidirectional | 1015 | down |
| uc003tvo.2 | 1.5130222 | 0.006151646 | intronic antisense | 1015 | down |
| ENST00000391861 | 1.5342886 | 0.00716163 | intergenic | 903 | down |
| G30897 | 1.6031789 | 0.02079475 | intergenic | 293 | down |
| AK124812 | 1.8175893 | 0.042965747 | intronic antisense | 1860 | down |
| AK124812 | 1.8175893 | 0.042965747 | intronic antisense | 1860 | down |
| AK124812 | 1.8175893 | 0.042965747 | intronic antisense | 1860 | down |
| AK124812 | 1.8175893 | 0.042965747 | intronic antisense | 1860 | down |
| AK124812 | 1.8175893 | 0.042965747 | intronic antisense | 1860 | down |
| AK124812 | 1.8175893 | 0.042965747 | intronic antisense | 1860 | down |
| AK124812 | 1.8175893 | 0.042965747 | intronic antisense | 1860 | down |
| AK124812 | 1.8175893 | 0.042965747 | intronic antisense | 1860 | down |
| AK124812 | 1.8175893 | 0.042965747 | intronic antisense | 1860 | down |
| ENST00000427452 | 1.517337 | 0.02669766 | intergenic | 404 | down |
| uc001elu.1 | 1.6164951 | 0.03000402 | intergenic | 896 | down |
| ENST00000416133 | 1.9309021 | 0.013890778 | intergenic | 1724 | down |
| uc.333- | 1.6913692 | 0.045938022 | exon sense-overlapping | 270 | down |
| uc.481+ | 1.6468012 | 0.036090806 | exon sense-overlapping | 204 | down |
| uc.481+ | 1.6468012 | 0.036090806 | exon sense-overlapping | 204 | down |
| uc.481+ | 1.6468012 | 0.036090806 | exon sense-overlapping | 204 | down |
| uc.481+ | 1.6468012 | 0.036090806 | exon sense-overlapping | 204 | down |
| ENST00000438308 | 1.6763409 | 0.035698637 | intergenic | 1513 | down |
| ENST00000434827 | 1.5559744 | 0.021666024 | intergenic | 780 | down |
| ENST00000435542 | 1.6996889 | 0.022832472 | intergenic | 565 | down |
| BC021185 | 1.5161153 | 0.03141964 | intergenic | 390 | down |
| AK123205 | 1.5169533 | 0.018359821 | intronic antisense | 1634 | down |
| AK123205 | 1.5169533 | 0.018359821 | intronic antisense | 1634 | down |
| CR619240 | 1.8972642 | 0.029353099 | natural antisense | 786 | down |
| ENST00000420855 | 1.9435316 | 0.008820087 | intronic antisense | 572 | down |
| ENST00000401578 | 1.8813248 | 0.03777824 | intergenic | 3853 | down |
| uc003ill.2 | 1.503942 | 0.048776515 | intergenic | 2134 | down |
| AK126981 | 1.6596564 | 0.04102131 | intergenic | 3623 | down |
| ENST00000505191 | 1.5883518 | 0.01020806 | intergenic | 879 | down |
| CR603564 | 2.5069768 | 0.03529359 | intergenic | 1051 | down |
| BC031882 | 3.314694 | 0.047163285 | intergenic | 1174 | down |
| ENST00000514634 | 1.5331035 | 0.042305164 | intergenic | 987 | down |
| ENST00000328088 | 1.8077493 | 0.042435877 | intergenic | 1707 | down |
| ENST00000412860 | 1.7557901 | 0.048235964 | intergenic | 1610 | down |
| BC030748 | 1.7992355 | 0.009321267 | intergenic | 1343 | down |
| ENST00000479244 | 1.5574203 | 0.014087539 | intergenic | 705 | down |
| AK125173 | 1.8805395 | 0.017347611 | natural antisense | 3232 | down |
| ENST00000436250 | 1.7400285 | 0.03229642 | intergenic | 252 | down |
| ENST00000485162 | 1.5619752 | 0.012986187 | intronic antisense | 587 | down |
| ENST00000485162 | 1.5619752 | 0.012986187 | intronic antisense | 587 | down |
| ENST00000485162 | 1.5619752 | 0.012986187 | intronic antisense | 587 | down |
| ENST00000485162 | 1.5619752 | 0.012986187 | natural antisense | 587 | down |
| ENST00000485162 | 1.5619752 | 0.012986187 | natural antisense | 587 | down |
| ENST00000485162 | 1.5619752 | 0.012986187 | intronic antisense | 587 | down |
| ENST00000485162 | 1.5619752 | 0.012986187 | bidirectional | 587 | down |
| ENST00000405526 | 1.8951722 | 0.04871701 | intergenic | 789 | down |
| uc004abd.1 | 1.6771567 | 0.040039055 | intergenic | 1713 | down |
| AK097380 | 1.6597627 | 0.028762424 | intergenic | 1759 | down |
| ENST00000425161 | 1.5999821 | 0.021023167 | natural antisense | 358 | down |
| ENST00000425161 | 1.5999821 | 0.021023167 | natural antisense | 358 | down |
| uc003weh.1 | 1.5218362 | 0.018543506 | intergenic | 2254 | down |
| uc001jef.2 | 2.211655 | 0.044103086 | intergenic | 2876 | down |
| uc003wrb.1 | 1.5184376 | 0.007865962 | intergenic | 707 | down |
| NR_028477 | 1.5599023 | 0.028217975 | exon sense-overlapping | 2132 | down |
| NR_028477 | 1.5599023 | 0.028217975 | exon sense-overlapping | 2132 | down |
| uc003pyw.1 | 2.1060965 | 0.043887947 | intergenic | 2435 | down |

“FCAbsolute”: fold change, the absolute ratio (no log scale) of normalized intensities between two conditions (cancer/para-cancer value in “up regulation” group and para-cancer/cancer value in “down regulation” group); “*P*-value”: calculated from paired t-test; “Relationship”: the relationship of lncRNA and its nearby coding gene and the coordinate of the coding gene.

"sense overlapping": the lncRNA's exon is overlapping a coding transcript exon on the same genomic strand; "intronic": the lncRNA is overlapping the intron of a coding transcript on the same genomic strand; "natural antisense": the lncRNA is transcribed from the antisense strand and overlapping with a coding transcript; "non-overlapping antisense": the lncRNA is transcribed from the antisense strand without sharing overlapping exons; "bidirectional": the lncRNA is oriented head to head to a coding transcript within 1000 bp; "intergenic": there are no overlapping or bidirectional coding transcripts nearby the lncRNA.

**Table S5: The mass spectrometry analysis of the proteins pulled down by *lincRNA-APOC1P1-3***

| **Accession** | **Score** | **Description (name)** | **Queries matched** |
| --- | --- | --- | --- |
| gi\|374822 | 235 | Chain B, Tubulin Alpha-Beta Dimer, electron diffraction | 24 |
| gi\|223646838 | 233 | Tubulin beta-1 chain [Salmo salar] | 24 |
| gi\|7106439 | 232 | Tubulin beta-5 chain [Mus musculus] | 24 |
| gi\|47221548 | 232 | unnamed protein product [Tetraodon nigroviridis] | 24 |
| gi\|74204140 | 232 | unnamed protein product [Mus musculus] | 24 |
| gi\|74223737 | 232 | unnamed protein product [Mus musculus] | 24 |
| gi\|18088719 | 232 | Tubulin, beta [Homo sapiens] | 24 |
| gi\|38488747 | 232 | Tubulin, beta 5 [Danio rerio] | 24 |
| gi\|55742495 | 232 | Tubulin beta chain [Xenopus (Silurana) tropicalis] | 24 |
| gi\|338695 | 232 | Beta-tubulin [Homo sapiens] | 24 |
| gi\|224473824 | 232 | beta-tubulin [Oryzias dancena] | 24 |
| gi\|57491330 | 232 | GekBS060P [Gekko japonicus] | 24 |
| gi\|135490 | 232 | Tubulin beta chain; | 24 |
| gi\|67463742 | 232 | Chain B, Tubulin-Colchicine-Vinblastine | 24 |
| gi\|194881355 | 230 | GG20906 [Drosophila erecta] | 24 |
| gi\|239582731 | 230 | tubulin, beta 2c [Danio rerio] | 24 |
| gi\|37681963 | 230 | tubulin, beta, 2 [Danio rerio] | 24 |
| gi\|223647034 | 230 | Tubulin beta-2C chain [Salmo salar] | 24 |
| gi\|295314924 | 230 | tubulin beta 1 [Hypophthalmichthys molitrix] | 24 |
| gi\|298351865 | 227 | Tubulin beta-2C chain | 21 |
